# ﻿Taxonomy of *Thelidiumauruntii* and *T.incavatum* complexes (lichenized Ascomycota, Verrucariales) in Finland

**DOI:** 10.3897/mycokeys.96.98738

**Published:** 2023-03-08

**Authors:** Juha Pykälä, Annina Kantelinen, Leena Myllys

**Affiliations:** 1 Nature solutions, Finnish Environment Institute, Latokartanonkaari 11, 00790, Helsinki, Finland Nature solutions, Finnish Environment Institute Helsinki Finland; 2 Botany Unit, Finnish Museum of Natural History, P.O. Box 7, FI-00014, University of Helsinki, Helsinki, Finland University of Helsinki Helsinki Finland

**Keywords:** Calcareous rocks, DNA barcoding, ITS, lichenized fungi, new species, phylogeny

## Abstract

The taxonomy of lichen species morphologically similar to *Thelidiumauruntii* and *T.incavatum* in Finland is being revised. Based on ITS and morphology, ten species occur in Finland. All species are restricted to calcareous rocks. The *Thelidiumauruntii* morphocomplex includes six species: *T.auruntii*, *T.huuskonenii***sp. nov.**, *T.pseudoauruntii***sp. nov.**, *T.sallaense* sp. nov, *T.toskalharjiense***sp. nov.** and *T.* sp. 1. In the ITS phylogeny, *T.auruntii*, *T.pseudoauruntii* and *T.sallaense* group together, but the remaining species are placed outside of this clade. All the species have northern distribution in Finland, occurring on fells in NW Finland and/or in gorges in the Oulanka area in NE Finland. The *Thelidiumincavatum* morphocomplex includes four species: *T.declivum***sp. nov.**, *T.incavatum*, *T.mendax***sp. nov.** and *T.* sp. 2. This morphogroup is not resolved as monophyletic in the ITS phylogeny, with only *T.declivum* and *T.mendax* forming a strongly supported group. *Thelidiumincavatum* is rather common in SW Finland, with one separate locality in eastern Finland. *Thelidiumdeclivum* occurs only in the Oulanka area. *Thelidiummendax* occurs in the Oulanka area, but one locality is known from eastern central Finland. *Thelidium* sp. 2 is known from one locality in SW Lapland.

## ﻿Introduction

Recently, many new species of *Verrucaria*, *Polyblastia* and related genera have been described from calcareous rocks of Europe (Savíc and Tibell 2008, 2012; Breuss and Berger 2012; Orange 2014, 2020; Tibell and Tibell 2015; Pykälä and Myllys 2016; Pykälä et al. 2017a, b, 2018, 2019, 2020). However, during that time, only one new species of *Thelidium* has been described from Europe (Ceynowa-Giełdon 2007). Thüs and Nascimbene (2008) revised the taxonomy of the Central European freshwater species of *Thelidium*. No recent study exists on the taxonomy of any species complexes of *Thelidium* on calcareous rocks.

*Thelidium* A. Massal. is a polyphyletic genus widely dispersed within the Verrucariaceae (Gueidan et al. 2007, 2009). The core of the species belongs to the so-called *Thelium* group, which also includes several species included in *Polyblastia* and *Verrucaria*.

In a previous study, we investigated the taxonomy of species of *Verrucaria* belonging to the *Thelidium* group and producing perithecia that leave pits on rocks in Finland (Pykälä et al. 2020). Here, we continue the study on the taxonomy of the *Thelidium* group, focussing on the species morphologically similar to *Thelidiumauruntii* (A. Massal.) Kremp. and *T.incavatum* Nyl. & Mudd. They are referred to here as *T.auruntii* and *T.incavatum* morphocomplexes. The *Thelidiumauruntii* complex is characterised by 1-septate spores, an involucrellum (predominantly short), pale, thin or endolithic thallus and medium-sized perithecia. The *Thelidiumincavatum* complex is characterised by 3-septate spores, perithecia leaving pits, medium-sized exciple (0.2–0.4 mm) and usually an endolithic pale thallus. Species belonging to the two complexes have similar habitat requirements and are restricted to calcareous rocks. Another species on calcareous rocks with 3-septate spores and perithecia leaving pits, *T.fontigenum* A. Massal., differs in its usually partly K+ violet thallus, smaller exciple (0.15–0.25 mm) and thin involucrellum.

## ﻿Methods

This study is based on the material collected by the first author during the lichen inventory of calcareous rocks and lime quarries in Finland in 2003–2011, supplemented by one specimen collected in 2018. The sampling was most extensive in southern Finland (over 50% of all calcareous rocks and lime quarries studied) (Pykälä et al. 2017a, b). The type material of putatively morphologically similar *Thelidium* species from herbaria B, H, H-NYL, M, PRM, S, UPS, VER were studied for comparison.

### ﻿Morphology

Perithecia and thalli were handsectioned with razor blades. The sections were examined and measured in tap water. Asci and ascospores were also studied in squash preparations of perithecia mounted in water. Sections and squash preparations of old herbarium specimens were studied using potassium hydroxide (KOH). Additionally, involucrellum characters and exciple colour and diameter were examined by cutting perithecia into two pieces and studying the pieces using a binocular microscope.

The range of spore size is indicated as arithmetic mean and standard deviation. Minimum and maximum values are given in parentheses. The size of the perithecia (in diameter) is given in surface view. The colour of the wall of the exciple was assessed from the basal parts.

### ﻿DNA extraction and sequencing

Total genomic DNA was extracted from perithecia (1–3) of two- to ten-year-old herbarium specimens. The samples were placed in 96-well microplates and sent to the Canadian Centre for DNA Barcoding (CCDB). CCDB’s standard protocols (documentation available at https://ccdb.ca/resources) were used for extraction, PCR and sequencing. Primers ITS1 and ITS4 (White et al. 1990) were used both for PCR and sequencing of the nuclear ribosomal ITS regions. The barcode sequences, their trace files along with all relevant collection data and photographs of the voucher specimens were uploaded to the Barcode of Life Data Systems (BOLD, https://www.boldsystems.org) database. The sequences are available in GenBank (see Table 1 for accession numbers).

**Table 1.** Specimens used in the phylogenetic analyses. New sequences are in bold.

Species	Country	Voucher	GenBank accession numbers
*Polyblastiaabscondita*	Sweden	Tibell 23641 (UPS)	EU553507
*P.albida*	Sweden	Savíc 3021 (UPS)	EU553492
*P.clandestina*	Sweden	Nordin 5466 (UPS)	EU559740
*P.lutosa*	Sweden	Savíc 3163 (UPS)	EU559734
*P.moravica*	Sweden	Savíc 3154 (UPS)	EU553522
*P.fuscoargillacea*	Sweden	Palice 7666 (hb. Palice)	EU553498
*P.* sp.	Sweden	Tibell 23635 (UPS)	EU553503
	Sweden	Savíc 3164 (UPS)	EU553519
***Thelidiumauruntii***	**Finland**	**Pykälä 36339 (H)**	**OP901843**
	**Finland**	**Pykälä 43414 (H)**	**OP901844**
	**Finland**	**Pykälä 43446 (H**	**OP901845**
	**Finland**	**Pykälä 43470 (H)**	**OP901846**
	**Finland**	**Pykälä 43829 (H)**	**OP901847**
	**Finland**	**Pykälä 43905 (H)**	**OP901848**
	**Finland**	**Pykälä 45171 (H)**	**OP901849**
***T.declivum***	**Finland**	**Pykälä 35996 (H)**	**OP901850**
	**Finland**	**Pykälä 36334 (H)**	**OP901851**
	**Finland**	**Pykälä 39640 (H)**	**OP901852**
	**Finland**	**Pykälä 39780b (H)**	**OP901853**
	**Finland**	**Pykälä 39997 (H)**	**OP901854**
	**Finland**	**Pykälä 40037 (H)**	**OP901855**
	**Finland**	**Pykälä 40047 (H)**	**OP901856**
	**Finland**	**Pykälä 44554 (H)**	**OP901857**
	**Finland**	**Pykälä 45123 (H)**	**OP901858**
***T.huuskonenii***	**Finland**	**Pykälä 31576 (H)**	**OP901859**
	**Finland**	**Pykälä 43243 (H)**	**OP901860**
	**Finland**	**Pykälä 43246 (H)**	**OP901861**
	**Finland**	**Pykälä 44167 (H)**	**OP901862**
***T.incavatum***	**Finland**	**Pykälä 34722 (H)**	**OP901863**
	**Finland**	**Pykälä 35282 (H)**	**OP901864**
	**Finland**	**Pykälä 36857 (H)**	**OP901865**
	**Finland**	**Pykälä 36867 (H)**	**OP901866**
	**Finland**	**Pykälä 37971 (H)**	**OP901867**
	**Finland**	**Pykälä 38227 (H)**	**OP901868**
	**Finland**	**Pykälä 38399 (H)**	**OP901869**
	**Finland**	**Pykälä 42871 (H)**	**OP901870**
	**Finland**	**Pykälä 46459 (H)**	**OP901871**
***T.mendax***	**Finland**	**Pykälä 39179 (H)**	**OP901872**
	**Finland**	**Pykälä 40152 (H)**	**OP901873**
	**Finland**	**Pykälä 42502 (H)**	**OP901874**
	**Finland**	**Pykälä 42503 (H)**	**OP901875**
*T.methorium*	Austria	Orange 21167 (NMW)	MT127220
	UK	Orange 16554 (NMW)	FJ645267
*T.papulare*	UK	Orange 16531 (NMW)	MT127197
*T.pertusatii*	Italy	Nascimbene JN1990	EU249471
***T.pseudoauruntii***	**Finland**	**Pykälä 45371 (H)**	**OP901876**
	**Finland**	**Pykälä 45374 (H)**	**OP901877**
*T.pyrenophorum*	Sweden	Tibell 23649 (UPS)	EU553500
***T.sallaense***	**Finland**	**Pykälä 44902 (H)**	**OP901878**
***T.toskalharjiense***	**Finland**	**Pykälä 43090 (H)**	**OP901879**
	**Finland**	**Pykälä 43211 (H)**	**OP901880**
	**Finland**	**Pykälä 43364 (H)**	**OP901881**
	**Finland**	**Pykälä 43398 (H)**	**OP901882**
	**Finland**	**Pykälä 43848 (H)**	**OP901883**
	**Finland**	**Pykälä 43861 (H)**	**OP901884**
*T.umbilicatum*	Sweden	Tibell 23525 (UPS)	EU559737
***T.* sp. 1**	**Finland**	**Pykälä 43208 (H)**	**OP901885**
***T.* sp. 2**	**Finland**	**Pykälä 52038 (H)**	**OP901886**
*Verrucariaaethiobola*	Norway	Orange 18946 (NMW)	MT127203
*V.anziana*	Norway	Orange 17221 (NMW)	FJ664835
*V.bifurcata*	Finland	Pykälä 36722 (H)	MT229720
”*V.calkinsiana*”	Canada	McMullin (OAC)	KT695332
*V.cavernarum*	Finland	Pykälä 37975 (H)	MT229724
*V.devergens*	Finland	Pykälä 45367 (H)	MT229741
”*V.deversa*”	Sweden	Savíc 3063 (UPS)	EU553496
*V.difficilis*	Finland	Pykälä 39060 (H)	MT229743
*V.karelica*	Finland	Pykälä 40325 (H)	MT229762
*V.kuusamoensis*	Finland	Pykälä 44694 (H)	MT229774
*V.latebrosa*	UK	Orange 16309 (NMW)	FJ664864
*V.pallidomurina*	UK	Orange 22835 (NMW)	MT127221
*V.subdevergens*	Finland	Pykälä 44550 (H)	MT229782
*V.subtilis*	Finland	Pykälä 37329 (H)	MT229810
*V.tephromela*	UK	Orange 19135 (NMW)	MT127212
*V.vacillans*	Finland	Pykälä 43272 (H)	MT229831
*V.* **sp.**	Norway	Orange 18952 (NMW)	MT127205

### ﻿Phylogenetic analyses

We searched for the closest relatives of the new *Thelidium* species by using the BLAST search facility in Genbank (https://blast.ncbi.nlm.nih.gov/Blast.cgi). Based on BLAST, all species belong to the *Thelidium* group, but most of them do not have close relatives in GenBank. We used the following criteria for including GenBank sequences in our phylogeny: two sequences per species were selected if they have more than 95% similarity to any of our study species. Additionally, one sequence per species bearing more than 90% similarity to any study species was selected if they were among the 100 most similar sequences according to BLAST.

Based on this search, six ITS sequences of *Polyblastia*, six sequences of *Thelidium* and 17 sequences of *Verrucaria* in GenBank were selected to reconstruct a putative phylogeny of the species (Table 1). *Polyblastiaalbida* Arnold and *P.fuscoargillacea* Anzi were used as outgroups based on the studies of Gueidan et al. (2009) and Pykälä et al. (2020).

A total of 76 ITS sequences were aligned with MUSCLE v.3.8.31 (Edgar 2004) using EMBL-EBI’s web service (https://www.ebi.ac.uk/Tools/msa/muscle/). The aligned data set was subjected to maximum likelihood analysis (ML). The analysis was performed with RAxML v.8.1.3 (Stamatakis 2014) located at CSC – IT Center for Science (https://www.csc.fi/english). The ITS region was partitioned into ITS1, 5.8S and ITS2. The GTRGAMMA model was used for all partitions. Node support was estimated with 1000 bootstrap replications using the rapid bootstrap algorithm.

## ﻿Results

**Figure 1.**
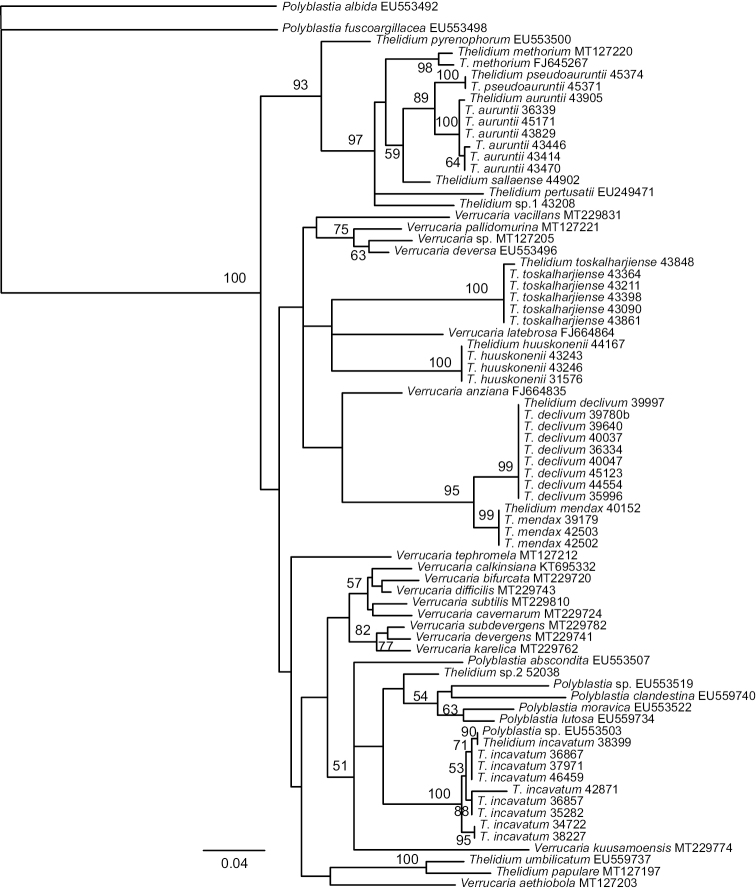
Phylogenetic relationships of the studied species. Strict consensus based on ITS data set with bootstrap values (>50%) at nodes. New species described in this study are indicated in bold.

We generated 44 new ITS sequences for this study (Table 1). The alignment consisted of 526 sites of which sites 1-193 belonged to the ITS1 rDNA regions, sites 194–345 to the 5,8S rDNA gene and sites 346–526 to the ITS2 rDNA regions. The alignment is available in the Suppl. material 1. In the ITS phylogeny, eight new lineages are observed (Fig. 1). The lineages, when represented by multiple samples, received high support values (99–100%). We describe six of these as new species: 1) *T.huuskonenii* sp. nov., represented by four specimens, 2) *T.pseudoauruntii* sp. nov. with two specimens, 3) *T.sallaense* sp. nov., with one specimen 4) *T.toskalharjiense* sp. nov., including six specimens, 5) *T.declivum* sp. nov., with nine specimens and 6) *T.mendax* sp. nov., with four specimens. Based on morphological characters, the first four species belong to the *T.auruntii* complex, while *T.declivum* and *T.mendax* are members of the *T.incavatum* complex. The two remaining lineages, both represented by only one specimen, are named here as *Thelidium* sp. 1 and *Thelidium* sp. 2. *Thelidium* sp. 1 resembles *T.auruntii*, whereas *Thelidium* sp. 2 is morphologically similar to *T.incavatum*. Both lineages most likely represent new species, although more specimens with similar morphology are needed before their status can be discussed further.

Based on the ITS phylogeny, the study species belong to the *Thelidium* group in the sense of Gueidan et al. (2009). Neither of the two morphocomplexes is resolved monophyletic. Instead, the ingroup is divided into three main subclades. The first subclade is strongly supported (93%) and includes *T.methorium*, *T.pertusatii* and *T.pyrenophorum* in addition to the three species of the *T.auruntii* morphocomplex, i.e., T.*auruntii*, *T.pseudoauruntii* sp. nov. and *T.sallaense* sp. nov. An unnamed lineage *Thelidium* sp. 1 belongs to this clade as well. *Thelidiumauruntii* and *T.pseudoauruntii* form a strongly supported group (89%) and *T.sallaense* is resolved as basal to them, although with low support (59%).

The second subclade remains unsupported and includes nine species in addition to one unnamed *Verrucaria* obtained from Genbank. *Thelidiumdeclivum* sp. nov. and *T.mendax* sp. nov., both belonging to the *T.incavatum* morphocomplex, form a strongly supported clade within this group. The second group also includes two new species, *T.huuskonenii* and *T.toskalharjiense*, which morphologically resemble *T.auruntii* and its allies.

The remaining two lineages of the *T.incavatum* morphocomplex, i.e., *T.incavatum* and *Thelidium* sp. 2, are clustered in the third subclade. The subclade remains unsupported and includes also four *Polyblastia*, two unidentified *Polyblastia*, two *Thelidium* and nine *Verrucaria* species, all retrieved from GenBank.

The results show low infraspecific variation in the ITS sequences of the study species. All specimens of *T.declivum* (n = 9), *T.huuskonenii* (n = 4), *T.mendax* (n = 4) and *T.pseudoauruntii* (n = 2) have identical ITS sequences. Low variation (> 99% similarity) between all specimens is also found in other species with the exception of one specimen of *T.incavatum*, which has only 98.5% similarity with the other specimens belonging to the same species.

Most of the species are restricted to calcareous rocks of northern Finland. *Thelidiumhuuskonenii*, *T.toskalharjiense* and *T.* sp. 1 have been found only from the calcareous fells of Enontekiö in NE Finland; *T.declivum*, *T.pseudoauruntii* and *T.sallaense* only from the Oulanka area (parishes Kuusamo and/or Salla) in NE Finland. *Thelidiummendax* occurs in the Oulanka area with one locality in eastern central Finland. *Thelidiumauruntii* occurs both in Enontekiö and the Kuusamo-Salla area. *Thelidium* sp. 2 has been found from one locality in SW Lapland. *Thelidiumincavatum* is the only species with southern distribution in Finland, occurring widely in SW Finland. One separate locality is in central eastern Finland.

## ﻿Discussion

The dominance of northern distribution within the study species may explain why most of them have remained undescribed. Only a few *Thelidium* species have been described from northern Europe and none from the calcareous fells. Particularly the crustose lichen flora of calcareous rocks in the fells of northern Finland is rather poorly known (Pykälä 2010a). Pyrenocarpous lichens in the calcareous fells of Sweden and Norway are also poorly collected (Magnusson 1952). It remains to be studied whether our new species occur more widely in the Scandinavian Mountains and in other northern mountains.

Species restricted to calcareous fells and/or the Oulanka area may be particularly sensitive to climate change. Northern lichen species may not be directly limited by temperatures but may be poor competitors, sensitive to the increase of other species benefitting from increased temperatures, particularly vascular plants (Alatalo et al. 2017; Greiser et al. 2021). For such species, retarding the dispersal of more southern species to the north is needed as a conservation measure of northern species against climate change (Pykälä 2017).

A considerable number of new species in the study group are congruent with the previous studies of Verrucariales in Finland (Pykälä and Myllys 2016; Pykälä et al. 2019, 2020). This study and earlier studies suggest that the number of species in Verrucariales in northern Europe is much higher than is presently known. We could find previously published names for only two of the species. This suggests that Fennoscandian and Central European species composition of the group under study differs greatly from each other. Similar results have been obtained among other previously studied groups of Verrucariales (e.g., Savić and Tibell 2012; Pykälä et al. 2017a, b, 2019, 2020).

## ﻿Taxonomy

The species descriptions are based on the sequenced specimens collected in Finland.

### ﻿ Thelidium auruntii

Taxon classificationFungiVerrucarialesVerrucariaceae

(A. Massal.) Kremp., Denkschr. Kgl. Bayer. Bot. Ges. 2(4): 248 (1861).

E4287970-D2CC-5FDE-86B6-E19CE8CDA88B

Verrucaria auruntii A. Massal., Symmict. Lich. 77. (1855). Basionym.

#### Type.

[Italy] ad saxa dolomitica in opp. Auronzo (Col della Favola) (VER!).

#### Description.

Prothallus not visible. Thallus pale brownish grey to pale greyish brown, rarely medium brown, continuous to rimose, rarely areolate, up to 0.2 mm thick, in one specimen surrounded by one dark line, algal cells c. 6–11 μm. Perithecia 0.21–0.38 mm in diam., 1/2–3/4-immersed, not leaving pits to leaving shallow pits; c. 60–160 perithecia / cm^2^. Ostiole dark, plane to depressed, c. 20–50 μm wide, ostiolar depression up to 120 μm wide. Involucrellum apical, exceeding half of the exciple to rarely to the exciple base level, 30–80 μm thick, appressed to the exciple or to clearly diverging from it. Exciple 0.16–0.29 mm, wall pale to dark brown (usually brown), c. 17–20 μm thick. Periphysoids c. 20–40(–50) × 1.5–2 μm. Asci c. 62–72 × 26–32 μm, 8-spored. Ascospores 0–1-septate, (20.3–)24.5–26.9–29.2(–32.1) × (9.2–)10.6–11.5–12.4(–13.3) μm (n = 86).

#### Habitat and distribution.

The species occurs in northern Finland in Enontekiö, Kuusamo and Salla parishes on dolomite rock outcrops, boulders, stones and pebbles. Several collections come from the calcareous fells of Saana and Toskalharji in Enontekiö. In the biogeographical province Koillismaa, the species is very rare, and it has been confirmed only from two localities (one in Kuusamo and one in Salla). The species grows mostly on dry sun-exposed calcareous rocks. However, some localities on river and brook shores are probably periodically submerged.

#### Notes.

The Finnish specimens are included in *T.auruntii*, because no clear morphological differences between the type of *T.auruntii* and the Finnish specimens were found. However, the type has slightly shorter spores: 20–26 × 10–14 μm. The type of *T.auruntii* is from the Dolomites in northern Italy. It is possible that *T.auruntii* is an arctic-alpine species. Sequences from the Dolomites are needed to confirm whether the Finnish specimens are conspecific with *T.auruntii*.

Pykälä (2007) reported the species as *T.pyrenophorum* (Ach.) Körb. from Finland, but the specimens differ morphologically from *T.pyrenophorum* (type: Helvetia, H-ACH-685!). The type of *T.pyrenophorum* differs in larger perithecia: 0.35–0.7 mm. According to our ITS phylogeny, the two species are rather closely related (Fig. 1) if the GenBank specimen of *T.pyrenophorum* from Sweden is correctly identified. *Thelidiummethorium* (Nyl.) Hellb. is characterised by large perithecia, thick involucrellum, larger spores (mean 34 × 14 μm) and occurrence on siliceous rocks (Thüs and Nascimbene 2008; Orange 2013). *Thelidiumpertusatii* (Garov.) Jatta resembles *T.methorium* but has a very thick involucrellum (110–170 μm thick) (Thüs and Nascimbene 2008). The differences between closely related *T.pseudoauruntii* and *T.sallaense* are discussed below.

Several other *Thelidium* species are morphologically rather similar to *T.auruntii*. *Thelidiumincinctum* (Vain.) Vain. (Finland, Kuusamo, Porontimajärvi, 12.8.1867, F.Sileń (H!, H-NYL6941, syntypes)) differs in thicker involucrellum (70–80 μm thick), larger spores (25–38 × 12–15 μm) and occurrence on siliceous rock. It is only known from the type collection. *Thelidiumbubulcae* (A.Massal.) Arnold described from Italy (ad saxa ipocretacea (Preapura) M. Bolca (M. Colle), 1854, A.Massalongo (VER!); Veneto; ad saxa arenacea oppidi Bolca (M. Colle) in prov. Veronensi Massalongo, Anzi: Lich. Rar. Veneti 136 (UPS-L-686416!, syntypes?) is rather similar to *T.auruntii* but may possibly differ by shorter, apical involucrellum and areolate thallus. *Thelidiumlacromense* Zschacke ([Croatia] ins. Lacroma: An frei liegenden Gestein, c. 10 m, Kalk, 11.9.1907, A.Latzel (PRM-858048!, syntype)) is morphologically similar to *T.auruntii*, but the type locality is close to sea level in Croatia and thus not likely to belong to the same species as the Finnish material of *T.auruntii*. *Thelidiumathallinum* Servít (Slovakia, Vysoké Tatry, monte Tokárna, supra saxa conglom., 1100–1200 m, 1925, Suza (PRM-858571!, syntype)) differs in thicker involucrellum (c. 80–100 μm thick).

#### Other specimens examined.

Finland, Koillismaa, Salla, Oulanka National Park, 400 m N of Savilampi, shore of river Savinajoki, dolomite rock outcrop, on NE-slope, 177 m a.s.l, 66°25'N, 29°10'E, 13 August 2009, J.Pykälä 36339 (H); Kuusamo, Oulanka National Park, Mataraniemi, shore of Oulankajoki river, treeless stony river shore, on dolomite stones, 145 m a.s.l, 66°22'N, 29°20'E, 26 August 2011, J.Pykälä 45171 (H); Enontekiön Lappi, Enontekiö, Porojärvet, Toskalharji, Toskaljärvi N, fell, dried old brook bottom, stony bottom, on dolomite pebbles, 707 m a.s.l., 69°11'N, 21°26'E, 3 August 2011, J.Pykälä 43414 (H); Enontekiö, Porojärvet, Toskalharji, Toskaljärvi N, fell, brook, W-shore, dolomite rock, outcrop, on N-slope, 710 m a.s.l, 69°11'N, 21°26'E, 3 August 2011, J.Pykälä 43446 (H); Enontekiö, Porojärvet, Toskalharji, Toskaljärvi N, fell, brook, W-shore, dolomite rock outcrop, on dolomite stones, 710 m a.s.l, 69°11'N, 21°26'E, 3 August 2011, J.Pykälä 43470 (H); Enontekiö, Porojärvet, Toskalharji, Toskaljärvi N, gentle SE-slope, alpine grassland, on dolomite boulder, 705 m a.s.l, 69°11'N, 21°26'E, 3 August 2011, J.Pykälä 43829 (H); Enontekiö, Kilpisjärvi, Saana, nature reserve, W-part, fell, dolomite rock outcrop, on SW-facing wall, 695 m a.s.l, 69°03'N, 20°48'E, 9 August 2011, J.Pykälä 43905 (H).

### ﻿ Thelidium declivum

Taxon classificationFungiVerrucarialesVerrucariaceae

Pykälä & Myllys sp. nov.

D80CDC61-6211-58AB-9ABF-A312FD20694E

846775

Figs 2A , 3H, I

#### Diagnosis.

Differing from *T.incavatum* by the perithecia often less immersed in rock, the involucrellum present in most perithecia and the exciple wall is thicker, most difficult to separate from *T.mendax* but the involucrellum on average is shorter.

#### Type.

Finland, Koillismaa, Salla, Oulanka National Park, 400 m N of Savilampi, shore of river Savinajoki, dolomite rock outcrop, on NE-slope, scarce, 178 m a.s.l, 66°25'N, 29°10'E, 13 August 2009, J.Pykälä 36334 (H-9204893, holotype, UPS, isotype, GenBank accession number: OP901851).

#### Description.

Prothallus not visible. Thallus white, grey or pale brown, endolithic to rarely thinly epilithic, algal cells c. 5–8 μm, in one specimen (type) surrounded by dark lines. Perithecia 0.12–0.44 mm in diam., 1/2–1-immersed, often surrounded by thalline collar, leaving shallow to usually deep pits; c. 20–80 perithecia / cm^2^. Ostiole pale to dark, plane to depressed, c. 20–50 μm wide. Involucrellum absent, apical or covering half of the exciple, 0–70 μm thick, appressed to the exciple or clearly diverging from it. Exciple 0.22–0.38 mm, wall dark brown to black, c. 25–40 μm thick, apex may be thickened to 50–70 μm thick. Periphysoids c. 30–50 × 1.5–2.5 μm. Asci c. 91–132 × 31–48 μm, 8-spored. Ascospores (1–2–)3-septate, few spores submuriform with 5–6(–8) cells, (32.3–)35.3–37.8–40.4(–44.4) × (12.6–)14.0–15.0–16.1(–17.3) μm (n = 69).

**Figure 2.**
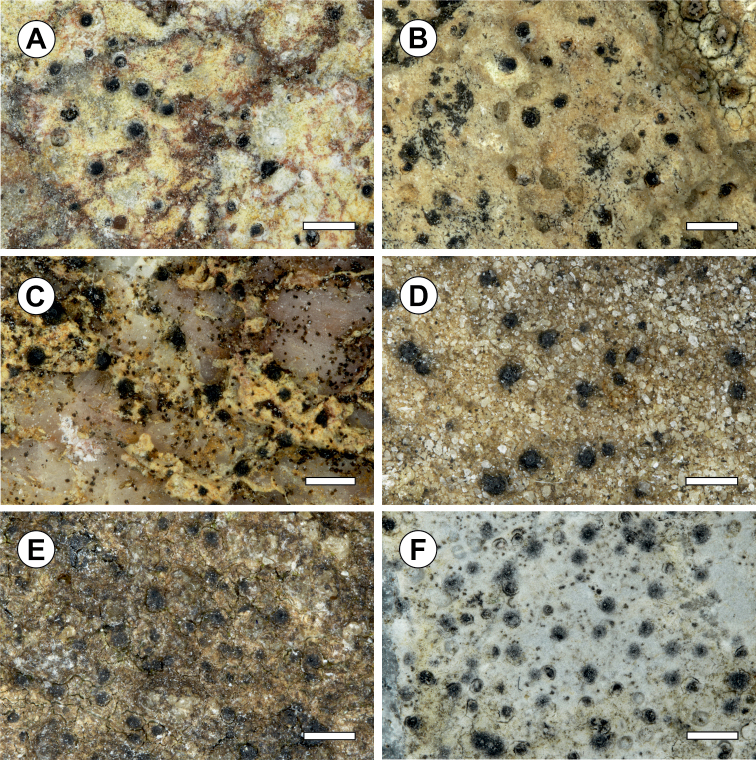
**A***Thelidiumdeclivum* (holotype) **B***T.huuskonenii* (holotype) **C***T.mendax* (holotype) **D***T.pseudoauruntii* (holotype) **E***T.sallaense* (holotype) **F***T.toskalharjiense* (holotype). Scale bars: 1 mm (**A–C, E, F**); 0.5 mm (**D**).

#### Habitat and distribution.

The species occurs in NE Finland, in the parishes of Kuusamo and Salla, on dolomite rock outcrops and boulders. The species may prefer rather shady habitats. Most localities are close to river shores.

#### Etymology.

The species prefers steep rock outcrops.

#### Notes.

This species was previously reported from Finland as *T.larianum* A. Massal. (Pykälä 2010b). The type specimen of *T.larianum* A. Massal. (in calcaris ubique, Garovaglio, VER!) is in poor condition, and thus, the identity of the species cannot be solved. According to Zschacke (1934), the spore size of *T.larianum* is smaller: 25–36 × 11–15 μm than the size in the Finnish specimens. The protologue (Massalongo 1855): “in circa Larium Lacum ad saxa calcareo-bituminosa Clar. Prof. Garov.”, i.e., the locality is close to Lake Como, and the collector is S. Garovaglio. Thus, the climatic conditions of the type locality are likely to strongly differ from those in NE Finland, and it is likely that the Finnish specimens do not belong to *T.larianum*. Based on the Finnish distribution, it could be assumed that *T.declivum* may be an eastern species, which has its main distribution in Russia and/or in North America.

*Polyblastiatorrentis* Servít (Sweden. Torne Lappmark: Jukkasjärvi par., Abisko, Regio subalpina by the torrent, alt. 400 m, 29.7.1921, A.H.Magnusson; UPS!, syntype) differs in less immersed perithecia (1/4–1/2-immersed) leaving shallow pits. Exciple may also be smaller (in studied perithecia c. 0.2–0.22 mm), but according to Servít (1953) the size of the exciple is c. 0.3 mm.

*Thelidiumdeclivum* is difficult to separate from *T.incavatum* and the closely related *T.mendax*. *Thelidiumincavatum* has predominantly fully immersed perithecia, and 3/4-immersed perithecia are usually overmature, whereas *T.declivum* has predominantly 3/4–immersed perithecia. Furthermore, the involucrellum is absent from most specimens of *T.incavatum*, whereas the involucrellum occurs in most perithecia, and in all studied specimens of *T.declivum*. *Thelidiumincavatum* also has a thinner exciple wall: c. 15–25 μm thick. *Thelidiummendax* may differ in longer involucrellum, but more material is needed to determine whether this character can be used to separate the species from *T.declivum*.

#### Other specimens examined.

Finland, Koillismaa, Kuusamo, Oulanka, Putaanoja, 500 m W-NW of Hautala, dolomite rock outcrop, beneath N-facing wall, on boulder, 228 m a.s. l., 66°22'N, 29°25'E, 9 August 2009, J.Pykälä 35996 (H); Salla, Oulanka National Park, Savikoski 300 m N, shore of lake Savilampi, calciferous (dolomite) schistose rock outcrop, on N-facing wall, 175 m a.s.l, 66°25'N, 29°10'E, 10 August 2010, J.Pykälä 39640 (H); Salla, Oulanka National Park, Savilamminniemi, shore of lake Savilampi, cliff, calciferous (dolomite) schistose rock outcrop, on S-facing wall, 172 m a.s.l, 66°25'N, 29°10'E, 12 August 2010, J.Pykälä 39780b (H); Kuusamo, Oulanka, Putaanoja, 500 m W-NW of Hautala, dolomite rock outcrop, on NE-facing wall, 230 m a.s.l., 66°22'N, 29°25'E, 15 August 2010, J.Pykälä 39997 (H), 40037 (H), 40047 (H); Kuusamo, Oulanka National Park, Taivalköngäs, shore of Oulankajoki river, *Piceaabies*-dominated herb-rich forest, dolomite rock outcrop, NE-slope, on dolomite boulder, 174 m a.s.l, 66°24'N, 29°11'E, 20 August 2011, J.Pykälä 44554 (H); Kuusamo, Oulanka National Park, Taivalköngäs, shore of Oulankajoki river, dolomite rock outcrop, on NW-facing wall, 165 m a.s.l, 66°24'N, 29°11'E, 25 August 2011, J.Pykälä 45123 (H).

### ﻿ Thelidium huuskonenii

Taxon classificationFungiVerrucarialesVerrucariaceae

Pykälä & Myllys sp. nov.

5F0E1016-7069-5D48-9CDC-4D80DB6C21A6

846776

Figs 2B , 3A, B

#### Diagnosis.

Species morphologically rather similar to *T.auruntii*, but the spores smaller, perithecia often leaving deep pits, and the thallus predominantly endolithic.

#### Type material.

***Holotype*.** Finland, Enontekiön Lappi, Enontekiö, Kilpisjärvi, Saana, fell, steep NE-slope, dolomite rock outcrop, on NE-facing wall, abundant, 820 m a.s.l, 69°02'N, 20°51'E, 11 August 2011, J.Pykälä 44167 (H9204900, GenBank accession number: OP901862).

#### Description.

Prothallus not visible. Thallus white to grey, endolithic to thinly epilithic, only surrounding perithecia, algal cells, c. 4–7 μm, in one specimen surrounded by one dark line. Perithecia 0.18–0.32 mm in diam., (1/2–)3/4–1-immersed, leaving shallow to deep pits; c. 80–120 perithecia / cm^2^. Ostiole pale to dark, plane to depressed, c. 20–40 μm wide. Involucrellum absent, apical or covering half of the exciple, 0–60 μm thick, appressed to the exciple or clearly diverging from it. Exciple 0.20–0.28 mm, wall dark brown to black, c. 15–20 μm thick, apex may be thickened to 50–80 μm thick. Periphysoids c. 20–45 × 1.5–2 μm, branching. Asci c. 58–74 × 22–25 μm, 8-spored. Ascospores (0–)1-septate, (16.4–)18.2–20.7–23.2(–27.6) × (8.0–)8.6–9.9–11.2(–14.4) μm (n = 69).

#### Habitat and distribution.

Four specimens have been confirmed by sequencing, but more than twenty morphologically similar specimens have been collected from the calcareous fells of Saana and Toskalharji. The species seems to occur only in northern Finland, in Enontekiö on fells on dolomite rock outcrops, boulders, stones and pebbles. It grows on southern and northern slopes and on rather flat surfaces. The altitudinal range is 705–890 m a.s.l.

**Figure 3.**
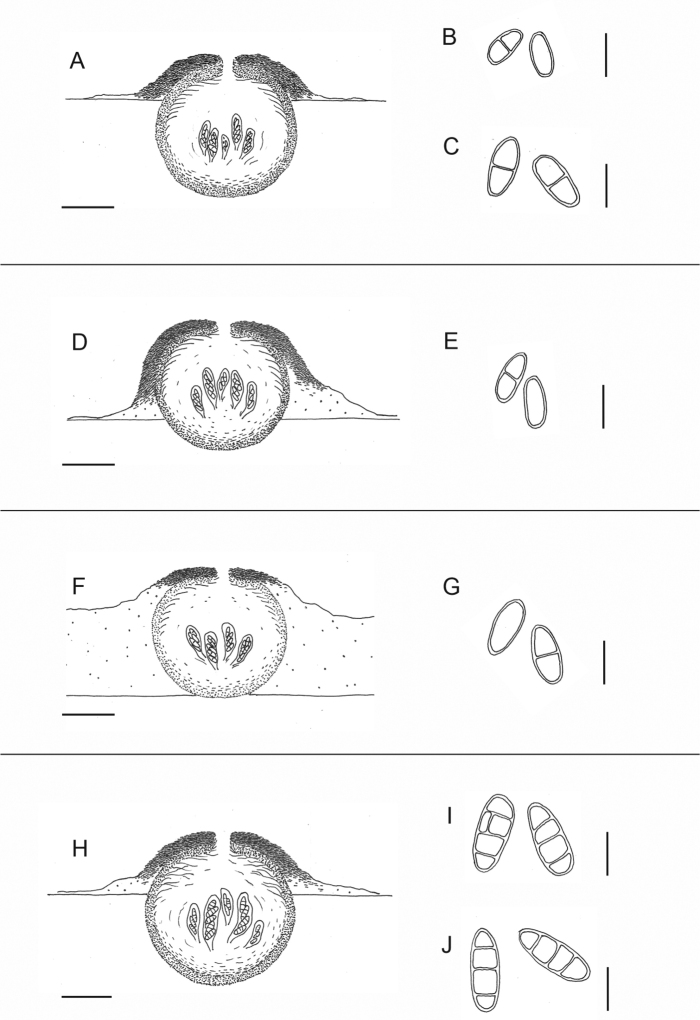
Drawn anatomical features of the newly described *Thelidium* species. Represented perithecial features are similar between *T.huuskonenii* and *T.toskalharjiense*, as well as between *T.declivum* and *T.mendax*; therefore, they are shown combined. Spores are represented as intermediate size for all species **A** perithecia of *T.huuskonenii* and *T.toskalharjiense* combined **B** spores of *T.huuskonenii***C** spores of *T.toskalharjiense***D** perithecium of *T.pseudoauruntii***E** spores of *T.pseudoauruntii***F** perithecium of *T.sallaense***G** spores of *T.sallaense***H** perithecia of *T.declivum* and *T.mendax* combined **I** spores of *T.declivum***J** spores of *T.mendax*. Scale bars for perithecia are 100 μm, and for spores 20 μm. Illustration: A. Kantelinen

#### Etymology.

The species is named after A.J.Huuskonen, the Finnish amateur lichenologist, who most intensively collected lichens from the Enontekiö area.

#### Notes.

The species differs from *T.auruntii* in smaller spores, perithecia often leaving deep pits and thinner, predominantly endolithic, thallus. The species has been previously reported from Finland as *T.decussatum* (Pykälä 2010a). The Finnish specimens have similar perithecia and involucrellum as *T.decussatum*. However, *T.decussatum* ([Germany] Trier, 1862, Mezler, Rabenhorst Lichenes Europaei 646 (UPS!, syntype)) has a rimose thallus and the spores may be slightly larger (20–25 × 10–11 μm). Furthermore, the type locality is in a temperate zone in lowland Germany. Phytogeographically, it is unlikely that such species would occur only in fells in the northwestern most part of Finland. *Thelidiumstenosporum* Servít (Slovakia: Bielské Tatry: in rup. calc. in convalle Dominův důl, … m. Havran … norg, 1700 m, 1937, Suza (PRM-858497!, syntype)) has smaller perithecia and narrower spores. *Thelidiumbubulcae* (A.Massal.) Arnold (syntypes: VER, UPS-L-686416!)) has larger rimose to areolate thallus, larger perithecia (0.2–0.37 mm) and larger spores: 20–27 × 9–13 μm. *Thelidiumpolycarpum* Servít (Montenegro, Lovčen, Ljubin potok, 1230 m, 1929, Servít (PRM-858491!, holotype)) has smaller perithecia (0.13–0.23 mm) and spores (15–23 × 8–9 μm). *Thelidiumklementii* Servít has rather thick rimose to areolate thallus and slightly larger spores and is only known from a temporarily inundated calcareous rock (Thüs and Nascimbene 2008).

#### Other specimens examined.

Finland. Enontekiön Lappi, Enontekiö, Kilpisjärvi, Saana fell, Saana nature reserve, low arctic zone, on SW-facing wall of dolomite rock outcrop, 880 m a.s.l., 69°02'N, 20°51'E, 27 July 2007, J.Pykälä 31576 (H); Enontekiö, Porojärvet, Toskalharji, Toskaljärvi N, fell, dolomite rock outcrop, on S-slope, 730 m a.s.l, 69°12'N, 21°26'E, 2 August 2011, J.Pykälä 43243 (H); Enontekiö, Porojärvet, Toskalharji, Toskaljärvi N, fell, dolomite rock outcrop, on dolomite pebbles, 735 m a.s.l, 69°12'N, 21°26'E, 2 August 2011, J.Pykälä 43246 (H).

### ﻿ Thelidium incavatum

Taxon classificationFungiVerrucarialesVerrucariaceae

Nyl. & Mudd, Man. Brit. Lich. 295 (1861)

2904BD03-D8C4-5017-94BF-AA95C28ADB28

= Thelidiumanisomerum Hellb., Kgl. Svensk. Vet. Akad. Handl. 9(11):34 (1871). Type. Sweden, Gotland, Linde klint, C.Stenhammar (S-L-347!); Gotland in colle calcareo ad Linde, Stenhammar 34 (S-L-250!), syntypes.
= Thelidiumdecipiescens Vain., Acta Soc. Fauna Flora Fenn. 49(2): 129 (1921). Type. Finland, Ab, Finby [= Varsinais-Suomi, Salo, Särkisalo], Förby, in rupe calcaria, 20 August 1920, E.Vainio (TUR-V!).
= ?Polyblastiasepulta A.Massal., Lotos 6: 81 (1856). Type: [Italy,] Circa Veronam ad saxa eocenica, A.Massalongo (VER!, syntype).

#### Type.

[England] 282. Bilsdale, Yorkshire, W.Mudd (M-0207325!, syntype?).

#### Description.

Prothallus not visible. Thallus white to grey, endolithic, in two specimens partly epilithic, slightly rimose, up to 0.1–0.15 mm thick, algal cells c. 4–6 μm. Perithecia 0.06–0.36 mm in diam., (1/2–)3/4–1-immersed, often thalline covered except apex, leaving deep pits; c. 20–160 perithecia / cm^2^. Ostiole pale to dark, plane, c. 20–40 μm wide. Involucrellum absent or apical, c. 30–60(–90) μm thick. Exciple 0.16–0.39 mm, wall dark brown to black, c. 15–25 μm thick, often with ostiolar neck, often pear-shaped. Periphysoids c. 40–50 × 1.5–2 μm. Asci c. 93–122 × 33–52 μm, 8-spored. Ascospores 3-septate, few spores submuriform with 5–7 cells, (32.3–)37.5–40.9–44.3(–49.4) × (12.2–)13.7–14.9–16.2(–18.1) μm (n = 78), rarely with a perispore c. 1 μm thick.

#### Habitat and distribution.

The species occurs in many localities in SW Finland on calcareous rocks and in lime quarries. It occurs on south- as well as on north-facing walls and on gentle slopes. More rarely, it grows on boulders, stones and pebbles. At present, more than 50 localities are known. Most localities are in the hemiboreal vegetation zone, a few in the southern boreal vegetation zone, only some tens of kilometres north of the limit of the hemiboreal zone. However, one separate locality occurs in Kuopio (former Juankoski) in eastern central Finland. *Thelidiumincavatum* may be the most common species of *Thelidium* in SW Finland, together with *T.minutulum* Körb.

#### Notes.

The observed syntype of *Polyblastiasepulta* is somewhat similar to *T.incavatum*, and *P.sepulta* may be the oldest name available for the species. However, better material of *P.sepulta* is needed to confirm it. Furthermore, it is uncertain whether *T.incavatum* is the correct name for the Finnish specimens. The syntype in M differs in rimose thallus. However, several localities have been included in the protologue, and the specimen in M may be untypical for *T.incavatum*. The Finnish specimens fit well with the description of *T.incavatum* by Orange (2013).

If the Finnish specimens do not belong to *T.incavatum*, there are two other putative younger names available for them. *Thelidiumdecipiescens* was described from SW Finland (Vainio 1921) and is morphologically similar to the sequenced Finnish specimens of *T.incavatum*. Already Vainio (1921) considered *T.decipiescens* to be similar to some material of *T.incavatum*. *Thelidiumanisomerum* Hellb., described from Sweden, is also similar to *T.incavatum*.

*Thelidiumbavaricum* Dalla Torre & Sarnth. has broader spores (Germany, Bavaria, an den aus den begrasten Boden hervorstehenden Dolomitblöcken oberhalb des Tiefenthales bei Eichstätt, April 1859, Arnold, in Arnold: Lich.. Exs. 87 (UPS-L-165297!, syntype): 30–48 × 15–20 μm, and submuriform spores may be more frequent.

The ITS sequence of the *T.incavatum* specimen, collected in the separate locality in eastern Finland, differs from the other sequenced specimens (98.5% similarity). The specimen also differs morphologically from the other specimens by less immersed perithecia (1/4–1/2-immersed) and thicker involucrellum (c. 70–90 µm thick). The spores may also be slightly shorter: 28.1–35.7 x 12.8–15.4 µm (n=15). It may represent a separate species but is now included in *T.incavatum*, awaiting more material.

#### Other specimens examined.

Finland, Varsinais-Suomi, Parainen (Korppoo), Åfvensår, Kälklot island, calcareous rock outcrop on shore of the Baltic Sea, E-slope, on pebbles, 5 m a.s.l., 60°18'N, 21°31'E, 27 July 2009, J.Pykälä 35282 (H); Salo (Särkisalo), Förby, 200 m S of lime factory, calcareous rock outcrop, on SW-slope, 60°05'N, 22°52'E, 8 September 2009, J.Pykälä 36857 (H); Salo (Särkisalo), Förby, 200 m S of lime factory, abandoned lime quarry, on 40 cm high NW-facing wall, 18 m a.s.l, 60°05'N, 22°52'E, 8 September 2009, J.Pykälä 36867 (H); Parainen (Iniö), Söderby, Biskopsö island, calcareous rock outcrop on shore of the Baltic Sea, on N-slope, 8 m a.s.l, 60°20'N, 21°28'E, 9 June 2010, J.Pykälä 37971 (H); Parainen (Korppoo), Elfsjö, Stora Limskär island, shallow abandoned lime quarry, on N-facing wall, 6 m a.s.l., 60°09'N, 21°26'E, 21 June 2010, J.Pykälä 38227 (H); Parainen (Houtskari), Björkö, Östra Långholm, Norrnäs, siliceous rock outcrop on shore of the Baltic Sea, NW-slope, on narrow calcareous vein, scarce, 9 m a.s.l., 60°15'N, 21°26'E, 29 June 2010, J.Pykälä 38399 (H); Parainen, Petteby, Kalkudden, abandoned lime quarry, on W-facing wall, 18 m a.s.l., 60°17'N, 22°10'E, 5 October 2011, J. Pykälä 46459 (H); Uusimaa, Hyvinkää, Myllykylä, Kalkkikallio, abandoned lime quarry, on N-facing wall, 100 m a.s.l, 60°36'N, 25°03'E, 7 July 2009, J.Pykälä 34722 & H. Rämä (H); Pohjois-Karjala, Kuopio (Juankoski), Siikajärvi, Huosiaisniemi, nature reserve, dolomite rock outcrop on shore of lake Ala-Siikajärvi, on gentle NE-slope, scarce, 97 m a.s.l., 63°12'N, 28°21'E, 26 July 2011, J.Pykälä 42871(H).

### ﻿ Thelidium mendax

Taxon classificationFungiVerrucarialesVerrucariaceae

Pykälä & Myllys sp. nov.

0449FAF0-386F-5C6E-A73D-8586B299C1DB

846777

Figs 2C , 3H, J

#### Diagnosis.

Species morphologically rather similar to *T.declivum* and *T.incavatum*, but the involucrellum is on average longer.

#### Type material.

***Holotype*.** Finland, Koillismaa, Salla, Oulanka National Park, Pikkuköngäs, shore of river Oulankajoki, high cliff, calciferous (dolomite) schistose rock outcrop, W-facing wall, on dolomite patch, 180 m a.s.l, 66°25'N, 29°08'E, 4 August 2010, J.Pykälä 39179 (H-9206536, GenBank accession number: OP901872).

#### Description.

Prothallus not visible. Thallus white to pale brown, endolithic to weakly rimose, algal cells, c. 4–7 μm. Perithecia 0.18–0.38 mm in diam., 1/2–1-immersed, often surrounded by thalline collar, leaving shallow to deep pits; c. 20–60 perithecia / cm^2^. Ostiole pale to dark, plane, c. 20–60 μm wide. Involucrellum apical to exceeding half of the exciple, c. 40–80 μm thick, appressed to the exciple or clearly diverging from it. Exciple 0.18–0.36 mm, wall dark brown to black, rarely pale, c. 20–30 μm thick. Periphysoids c. 40–60 × 1.5–2.5 μm. Asci c. 90–128 × 31–43 μm, 8-spored. Ascospores 3-septate, (34.8–)36.1–40.1–44.1(–51.0) × (11.8–)12.8–13.4–14.0(–14.9) μm (n = 48), perispore 1–1.5 μm thick in few spores.

#### Habitat and distribution.

The species is known from three localities, two in NE Finland, in the parishes Kuusamo and Salla, and one in Juuka in central eastern Finland. *Thelidiummendax* grows on calcareous rocks and calciferous (dolomite) schistose rocks. Two collection sites are affected by spring flooding and may periodically be submerged.

#### Etymology.

The species is easily misidentified.

#### Notes.

Based on the ITS phylogeny, this species is closely related to *T.declivum*. It is also morphologically difficult to separate from that species. *Thelidiummendax* has, on average, longer involucrellum than in *T.declivum*. *Thelidiummendax* is here kept separated from *T.declivum* because this solution received high support value in the ITS phylogeny (99%). Within the species *T.declivum* and *T.mendax* have identical ITS sequences and the species have a clear barcoding gap (3% difference).

*Polyblastiatorrentis* (Sweden. Torne Lappmark: Jukkasjärvi par., Abisko, Regio subalpina by the torrent, alt. 400 m, 29 July 1921, A.H.Magnusson (UPS!, syntype)) differs in less immersed perithecia (1/4–1/2-immersed) and thinner involucrellum (c. 30–40 μm thick).

#### Other specimens examined.

Finland, Koillismaa, Kuusamo, Juuma, Oulanka National Park, Jäkälävuoma, gorge, calciferous (dolomite) schistose rock outcrop, on bottom, shore of a pond, on dolomite coated stone, 200 m a.s.l., 66°15'N, 29°26'E, 16 August 2010, J.Pykälä 40152 (H); Pohjois-Karjala, Juuka, Petrovaara, Riihilahti S, calcareous rock outcrop, on W-facing wall, 190 m a.s.l, 63°09'N, 28°58'E, 13 July 2011, J.Pykälä 42502 (H), 42503 (H).

### ﻿ Thelidium pseudoauruntii

Taxon classificationFungiVerrucarialesVerrucariaceae

Pykälä & Myllys sp. nov.

5A9F617F-42DD-5307-9A7E-6AB986EAD76A

846779

Figs 2D , 3D, E

#### Diagnosis.

Species morphologically rather similar to *T.auruntii*, but the spores are smaller and the perithecia tend to be less immersed.

#### Type material.

***Holotype*.** Finland, Kollismaa, Kuusamo, Oulanka National Park, Mataraniemi W, shore of Oulankajoki river, small dolomite rock outcrop, on SE-slope, 147 m a.s.l, 66°22'N, 29°20'E, 28 August 2011, J.Pykälä 45374 (H9220350, GenBank accession number: OP901877).

#### Description.

Prothallus not visible. Thallus pale greyish brown to medium brown, endolithic to rimose, algal cells c. 5–7 μm, with one dark brown thalline line. Perithecia 0.18–0.36 mm in diam., 1/4–1/2-immersed, not leaving pits to leaving shallow pits; c. 50–140 perithecia / cm^2^. Ostiole dark, depressed, c. 20–50 μm wide. Involucrellum covering half of the exciple, c. 40–70 μm thick, appressed to clearly diverging from the exciple. Exciple c. 0.17–0.24 mm, wall pale brown to usually dark brown, c. 15–20 μm thick. Periphysoids c. 20–25 × 2 μm. Asci c. 64–83 × 22–32 μm, 8-spored. Ascospores 0–1-septate, (21.3–)23.9–25.1–26.2(–26.6) × (8.6–)9.2–10.1–11.0(–12.2) μm (n = 23), perispore 1 μm thick present in some spores.

#### Habitat and distribution.

The species is only known from the type locality on small dolomite rock outcrop on the rivershore. The locality is likely to be under water during spring floods. Potentially suitable habitats for the species may be very rare in Finland.

#### Etymology.

The species resembles morphologically *T.auruntii* and is also closely related to the species based on the ITS sequences.

#### Notes.

The species is closely related to *T.auruntii* and *T.sallaense*. Only two specimens are known and from the same locality. We prefer to treat *T.pseudoauruntii* as a species separate from *T.auruntii* because of high support value (100%) in the ITS phylogeny. Furthermore, there is a clear barcoding gap (3%) between the species. The species have also different distribution areas. *Thelidiumpseudoauruntii* seems to be absent from the calcareous fells of NW Finland, i.e., from the main distribution area of *T.auruntii* in Finland. *Thelidiumpseudoauruntii* may be an eastern species with the main distribution area in North America and/or Russia. The specimens of *T.pseudoauruntii* have smaller spores than in *T.auruntii* and *T.sallaense*. Furthermore, the perithecia are less immersed. However, more material is needed to determine whether the species differs unambiguously morphologically from the related species.

#### Other specimens examined.

Finland, Koillismaa, Kuusamo, Oulanka National Park, Mataraniemi W, shore of Oulankajoki river, small dolomite rock outcrop, on dolomite stones, 145 m a.s.l., 66°22'N, 29°20'E, 28 August 2011, J.Pykälä 45371 (H).

### ﻿ Thelidium sallaense

Taxon classificationFungiVerrucarialesVerrucariaceae

Pykälä & Myllys sp. nov.

EE2A0399-CB8B-5187-91F4-E0C5BF3B1C20

846780

Figs 2E , 3F, G

#### Diagnosis.

Species morphologically rather similar to *T.auruntii*, but the thalli may be more brown-pigmented.

#### Type material.

***Holotype*.** Finland, Koillismaa, Salla, Oulanka National Park, Savilampi 1,4 km NE, shore of Savinajoki river, river shore, on dolomite stone, 185 m a.s.l., 66°26'N, 29°11'E, 23 August 2011, J.Pykälä 44902 (H9220340, GenBank accession number: OP901878).

#### Description.

Prothallus not visible. Thallus pale brown to medium brown, rimose, algal cells c. 5–8 μm. Perithecia 0.21–0.28 mm in diam., 3/4(–1)-immersed in thallus, not leaving pits; c. 60–80 perithecia / cm^2^. Ostiole brown, plane to depressed, c. 20–60 μm wide. Involucrellum apical, c. 40–50 μm thick, slightly diverging from the exciple. Exciple c. 0.16–0.27 mm, wall pale to brown, K+ olive. Periphysoids c. 20–30 × 2 μm. Asci c. 67–83 × 25–32 μm, 8-spored. Ascospores (0–)1-septate, (26.3–)27.3–28.8–30.2(–31.1) × (12.0–)12.2–12.6–12.9(–13.4) μm (n = 12), perispore 1 μm thick present in some spores.

#### Habitat and distribution.

The species is only known from the type locality growing on dolomite stone on a rivershore. The species is likely to be very rare and threatened in Finland.

#### Etymology.

The name is according to the parish Salla from where the only known specimen has been collected.

#### Notes.

More material is needed to solve whether the species can be morphologically separated from *T.auruntii*. It may differ from *T.auruntii* by a more clearly brown pigmented thallus.

### ﻿ Thelidium toskalharjiense

Taxon classificationFungiVerrucarialesVerrucariaceae

Pykälä & Myllys sp. nov.

283BB1BF-30E6-583A-88D0-AAEB8BC08E65

846781

Figs 2F , 3A, C

#### Diagnosis.

Species morphologically rather similar to *T.auruntii*, but the spores are larger, and the involucrellum is, on average, shorter and thinner.

#### Type material.

***Holotype*.** Finland, Enontekiön Lappi, Enontekiö, Porojärvet, Toskalharji, Toskaljärvi N, fell, stony lake shore, on dolomite stones, 705 m a.s.l., 69°11'N, 21°26'E, 3 August 2011, J.Pykälä 43398 (H9206484, GenBank accession number: OP901882).

#### Description.

Prothallus not visible. Thallus grey to pale brownish grey, endolithic to thinly epilithic, rarely rimose, algal cells c. 5–8 μm, sometimes surrounded by dark lines. Perithecia 0.17–0.36 mm in diam., 1/2–3/4(–1)-immersed, leaving shallow to deep pits; c. 60–160 perithecia / cm^2^. Ostiole pale to dark, plane, c. 20–30(–60) μm wide. Involucrellum apical to covering half of the exciple, 20–50 μm thick, appressed to the exciple or strongly diverging from it. Exciple c. 0.15–0.24 mm, wall dark brown to black, c. 20–30 μm thick. Periphysoids c. 20–30 × 2–2.5 μm. Asci c. 73–83 × 23–29 μm, 8-spored. Ascospores 1-septate, (22.4–)26.8–29.1–31.3(–35.5) × (11.8–)12.5–13.4–14.2(–16.1) μm (n = 91).

#### Habitat and distribution.

The species occurs in the fell Toskalharji in northern Finland in Enontekiö within an area of three square kilometres and is divided into three subpopulations. It grows on dolomite boulders, stones and pebbles. Most specimens are from dolomite pebbles. It mostly grows on gentle slopes. The altitudinal range is 705–875 m a.s.l.

#### Etymology.

The species has been found only in the Toskalharji area.

#### Notes.

The species differs from *T.auruntii* by larger spores, but there is a considerable overlap in the spore size. The involucrellum is, on average, shorter and thinner, but few specimens of *T.auruntii* may have similar involucrellum to *T.toskalharjiense*. Nevertheless, based on ITS phylogeny, the species is not closely related to *T.auruntii*.

#### Other specimens examined.

Finland, Enontekiön Lappi, Enontekiö, Porojärvet, Toskalharji, Toskalpahta fell, SW-slope, scree, on dolomite pebbles, 790 m a.s.l, 69°11'N, 21°29'E, 1 August 2011, J.Pykälä 43090 (H); Enontekiö, Porojärvet, Toskalharji, Toskaljärvi N, fell, gentle SE-slope, on dolomite boulder, 715 m a.s.l, 69°11'N, 21°26'E, 2 August 2011, J.Pykälä 43211 (H); Enontekiö, Porojärvet, Toskalharji, Toskaljärvi N, fell, gentle SW-slope, dolomite rock outcrop, on dolomite pebbles, 715 m a.s.l, 69°11'N, 21°26'E, 3 August 2011, J.Pykälä 43364 (H); Enontekiö, Porojärvet, Toskalharji, Toskaljärvi N, close by lake shore, gentle NE-slope, alpine grassland on dolomite stone, 706 m a.s.l, 69°11'N, 21°26'E, 7 August 2011, J.Pykälä 43848 (H); Enontekiö, Porojärvet, Toskalharji, Toskaljärvi N, close by lake shore, alpine grassland, gentle SE-slope, dolomite rock outcrop, on dolomite pebbles, 706 m a.s.l, 69°11'N, 21°26'E, 7 August 2011, J.Pykälä 43861 (H).

### ﻿ Thelidium

Taxon classificationFungiVerrucarialesVerrucariaceae

sp. 1

6E675AB6-F452-52DF-816B-7ECBB13D2229

#### Description.

Prothallus not visible. Thallus whitish grey, endolithic. Perithecia 0.25–0.33 mm in diam., 1/4–1/2-immersed, leaving shallow pits; c. 50 perithecia / cm^2^. Ostiole, tiny, dark, plane. Involucrellum almost to the exciple base, 30–50 μm thick, may thicken to the base to c. 50–60 μm thick, slightly diverging from the exciple. Exciple c. 0.17–0.25 mm, wall dark brown. Periphysoids c. 25–30 × 2 μm. Asci 8-spored. Ascospores 0–1-septate, 23–28 × 10–12 μm.

#### Habitat and distribution.

The specimen has been collected from a dolomite rock outcrop on a fell in northern Finland in Enontekiö.

#### Notes.

The specimen probably represents an undescribed species belonging to the *T.auruntii* complex. However, the specimen is quite small, and thus, better material is needed.

#### Specimen examined.

Finland, Enontekiön Lappi, Enontekiö, Porojärvet, Toskalharji, Toskaljärvi N, fell, dolomite rock outcrop, on SW-slope, 715 m a.s.l., 69°11'N, 21°26'E, 2 August 2011, J.Pykälä 43208 (H).

### ﻿ Thelidium

Taxon classificationFungiVerrucarialesVerrucariaceae

sp. 2

2B823D4E-3535-5520-9AB0-17892816085E

#### Description.

Prothallus not seen. Thallus whitish grey, endolithic. Perithecia 0.17–0.26 mm, 3/4–immersed, leaving deep pits; c. 40–60 perithecia /cm^2^. Ostiole dark, plane to depressed, c. 20–40 µm wide. Involucrellum absent. Exciple c. 0.26–0.32 mm, wall black, apex thickened.

Spores 3-septate, 30–37 x 16–18 µm.

#### Habitat and distribution.

The only specimen has been collected from calcareous pebbles in a lime quarry spoil in SW Lapland.

#### Notes.

The specimen is small. It is morphologically similar to *T.incavatum* and probably closely related to that species, although the relationship remains unsupported. The species may be undescribed but a better specimen is needed for type material.

#### Specimen examined.

Finland, Kittilän Lappi, Kolari, Äkäsjokisuu, Mannajoki, former cement factory 200 m N-NW, lime quarry spoil, on calcareous pebbles, 165 m a.s.l, 67°27'N, 23°38'E, 16 August 2018, J.Pykälä 52038 (H).

## Supplementary Material

XML Treatment for Thelidium auruntii

XML Treatment for Thelidium declivum

XML Treatment for Thelidium huuskonenii

XML Treatment for Thelidium incavatum

XML Treatment for Thelidium mendax

XML Treatment for Thelidium pseudoauruntii

XML Treatment for Thelidium sallaense

XML Treatment for Thelidium toskalharjiense

XML Treatment for Thelidium

XML Treatment for Thelidium

## Supplementary materials

Supplementary material 1 The alignment Data type PDF file https://binary.pensoft.net/file/821127This dataset is made available under the Open Database License (http://opendatacommons.org/licenses/odbl/1.0/). The Open Database License (ODbL) is a license agreement intended to allow users to freely share, modify, and use this Dataset while maintaining this same freedom for others, provided that the original source and author(s) are credited.Juha Pykälä, Annina Kantelinen, Leena Myllys
