# Supplementary material for: ﻿Taxonomy of Thelidiumauruntii and T.incavatum complexes (lichenized Ascomycota, Verrucariales) in Finland
Source: MycoKeys. 2023 Mar 8;96:1–23. doi: 10.3897/mycokeys.96.98738 (PMC10210258; doi:10.3897/mycokeys.96.98738)
Supplement: Supplementary material 1 — The alignment [file mycokeys-96-001-s001.pdf]

```
#NEXUS

[! Generated by PhyDE 0.9971 ]
[Tue Jan 24 15:14:12 EET 2023]


BEGIN DATA;
DIMENSIONS NTAX=75 NCHAR=526;
FORMAT DATATYPE=DNA GAP=- MISSING=? ;

MATRIX
Polyblastia_albida_EU553492           CATTACCGAG-TTAGGGTCCTCTGGGCCCC-
ATCT-CCCACTC-TTGTCTACCTTGAAACGCTG-CTCGACGGGCCCGTCTCTGACCGGACCGC----
CGGGGATTCTGTCCCCGGCCCGCGCCCGCCGACGGCCACTTTTAAA-CTCTTGCAACCAT---
GTCTAAATCGAA----ACCAAATTATC-
AAAACTTTCAACAACGGATCTCTTGGTTCTGGCATCGATGAAGAACGCAGCGAAATGCGATAAGTAATGCGAA
TTGCAGAATTCCGCGAGTCATCGAATCTTTGAACGCACATTGCGCCCTTTGGCATTCCGAAGGGCATGCCTGT
TCGAGCGTCATTATCAACCCCCAAGCCCGGCTTGTCGTTGGATCC-CACCGTCGTCCCCCGGCGGTGGACC-
CGAAAGATAGTGGCAGAGCC-CGTGGGA--CCCTGGATGCAGCGAGCTTCTT-
CAGCATGCACCCGGACGGTCT-CC-CGGCTCCGTT-TGAACC--ATCAAT-TTCATCNNNNNNNNN
Polyblastia_fuscoargillacea_EU553498  CATTATCGAG-TCAGGGTCCTCTGGGCCCC-
ACCT-CCAACTC-TTGCTTATCTTAATACGTTG-CTTGACGGGCCTGTCTCTAACCGGACCGT----
CGGGGATTCTGTCCCCGGCTCGCGTCCGTCAACGACCACTTTTATA-TCCTTCCAACCAT---
GTCAAATCGAAA----ATAGAACTATT-
AAAACTTTCAACAACGGATCTCTTGGTTCTGGCATCGATGAAGAACGCAGCGAAATGCGATAAGTAATGCGAA
TTGCAGAATTCCGTGAGTCATCGAATCTTTGAACGCACATTGCGCCCTTTGGTATTCCGAAGGGCATGCCTGT
TCGAGCGTCATTATCAACCCTCAAGCCCGGCTTGCAGTTGGCTTC-CACCGGCGG-CGACCCCGGTGGATC-
CGAAAGATAATGGCAGAGTC-GGTGGGA--CCCTGGATGCAGCGAGCTTTCT--
AGCATGCACTTCAACGGTCG-TC-CGGCTCAGCC-TCAACCA-ATTTAT-TTCATCTNNNNNNNN
Polyblastia_clandestina_EU559740      CATTATCGAG-CTAGGGTCCTCTGGGCCCC-
ATCT-CCAACCC-TTGTCTACC-----ACGTTG-CCC--CGG---CGCTCCTGCCGGGGTGATT---
TGAAAATTT--------------------------C--TTTTTAAA-TTC-
TTCAACCATGTCGTCCTACGGGAGATTAAACAATGAATT-
AAAACTTTCAACAACGGATCTCTTGGTTCTGGCATCGATGAAGAACGCAGCGAAATGCGATAAGTAATGCGAA
TTGCAGAATTCCGTGAGTCATCGAATCTTTGAACGCACATTGCGCCCTTTGGTATTCCGAAGGGCATGCCTGT
TCGAGCGTCATTATCAACCCTCAAGCCCGGCTTGCTGTTGGGTCC-CACCGTTGG-CGACGGCGGTGGACC-
CGAAAGGTAATGGCAGAGTC-TGCGAGA--CCCTGGATGCAGCGAGCTTTTT-
CAGCACGCACCCTCACGGCTT-CG-CGGGGCTGTC-TCAACCACACAATATACAATCTGGTTGACC
Polyblastia_abscondita_EU553507       CATTACCGAG-TTAGGGTCCTCTGGGCCCC-
ATCT-CCCACCC-TTGTCTACC-----ACGTCG-CCC--CGG---CGCCTCTGCCGGGGCGACTTT-
TGAAAATTT--------------------------C--TTTTTAAA-TTC-
TTCAACCATGACGTCCTACGGGAGAAT-ACCAATCAGTT-
AAAACTTTCAACAACGGATCTCTTGGTTCTGGCATCGATGAAGAACGCAGCGAAATGCGATAAGTAATGCGAA
TTGCAGAATTCCGTGAGTCATCGAATCTTTGAACGCACATTGCGCCCTTTGGTATTCCGAAGGGCATGCCTGT
CCGAGCGTCATTATCAACCCTCAAGCCCGGCTTGCTGTTGGGTCT-CACCGCCGT-CGCCGACGGTGGACC-
CGAAAGATAATGGCAGAGTC-TGCGAGA--CCCTGGATGCAGCGAGCTTCTT-
CAGCACGCATCACAACGGCCT-CG-TGGCTCCGTC-TCCACCA----ACA-TTTATCTNNNNNNNN
Polyblastia_moravica_EU553522         CATTATCGAG-CTAGGGTCCTCTGGGCCCC-
ATCT-CCAACCC-TTGTCTACT-----ACGTTG-CCC--CGG---CGTTCCTGCCGGGGTGACTT--
TGAAAATTT--------------------------C-TTTTTTAAA-TTC-
TTTAACCATGTCGTCCTACGGGAGAGTAAACAATAAATT-
AAAACTTTCAACAACGGATCTCTTGGTTCTGGCATCGATGAAGAACGCAGCGAAATGCGATAAGTAATGCGAA
TTGCAGAATTCCGTGAGTCATCGAATCTTTGAACGCATATTGCGCCCTTTGGTATTCCGAAGGGCATGCCTGT
TCGAGCGTCATTATCAACCCTCAAGCCCGGCTTGCTGTTGGGTCC-CACCGTCGG-CGACGGCGGTGGACC-
CGAAAGATAATGGCAGAGTC-TGCGAGA--CCCTGGATGCAGCGAGCTTCCT-
CAGCACGCATCGTACGGATCTCTG--GGCGCTGTC-TTAACCG-ACAAAT-TTCATCNNNNNNNNN
Polyblastia_lutosa_EU559734           CATTATCGAG-CTAGGGTCCTCTGGGCCCC-
ATCT-CCAACCC-TTGTCTACC-----ACGTTG-CCC--CGG---CGTTCCAGCCGGGGTGACTT--
```

TGAAAATTT-------------------------C-TTTTTTAAA-TTC-
TTTAACCATGTCGTCCTACGGGAGAGCAAACAATGAATT-
AAAACTTTCAACAACGGATCTCTTGGTTCTGGCATCGATGAAGAACGCAGCGAAATGCGATAAGTAATGCGAA
TTGCAGAATTCCGTGAGTCATCGAATCTTTGAACGCACATTGCGCCCTTTGGTATTCCGAAGGGCATGCCTGT
CCGAGCGTCATTATCAACCCTCAAGCCCGGCTTGCTGTTGGGTCC-CACCGTCGG-CGACGGCGGTGGACC-
CGAAAGATAATGGCAGAGTC-TACGAGA-CCCCTGGATGCAGCGAGCTTCCT-
CAGCACGCATCGTAAGGGCCC-CG-GGGCGCTGTC-TCAACCG-ATAAAT-TTCATCTGGTTGACC
Thelidium_papulare_MT127197   CATTATCGAG-TTAGGGTCCCCTGGGCCCC-
ATCT-CCAACCC-TTGTCTACC-----ACGTCG-CCC--CGG---CCCTTGCGCCGGAGTGACTT--
GAAAAAATT-------------------------C--TTTTTAAA-
TTCTTTCAACCATGACGTCCTGCGGGAGAATATCAAATTTATT-
AAAACTTTCAACAACGGATCTCTTGGTTCTGGCATCGATGAAGAACGCAGCGAAATGCGATAAGTAATGCGAA
TTGCAGAATTCCGTGAGTCATCGAATCTTTGAACGCACATTGCGCCCTTTGGTATTCCGAAGGGCATGCCTGT
TCGAGCGTCATTATCAACCCTCAAGCCCGGCTTGCTGTTGGATCC-CACCGTTGG-CGACGGCGGCGGATC-
CGAAAGACAATGGCGGAGTC-CGCGGGA--CCCCGGATGCAGCGAGCTTCTT-
CAGCACGCATCCCGGCGTCCC-CG-GGGCTCGGTC-TTGACCA-ATCAACACACATCTGGTTGACC
Thelidium_umbilicatum_EU559737   CATTATCGAG-TTAGGGTCCTCTGGGCCCC-
ATCT-CCAACCC-TTGTCTACC-----ACGTCG-CCC--CGG---CCCTTGCGCCGGAGTGATTTT-
TAAAAATTT-------------------------C--TTTTTAAA-
TTCTTTCAACCATGACGTCCTACGGGAGAAG-ATCAATTAAGT-
AAAACTTTCAACAACGGATCTCTTGGTTCTGGCATCGATGAAGAACGCAGCGAAATGCGATAAGTAATGCGAA
TTGCAGAATTCCGTGAGTCATCGAATCTTTGAACGCACATTGCGCCCTTTGGTATTCCGAAGGGCATGCCTGT
TCGAGCGTCATTATCAACCCTCAAGCCCGGCTTGCTGTTGGATCC-CACCGTTGG-CGACGGCGGTGGATC-
CGAAAGACAATGGCGGAGTC-CGCGGGA--CCCTGGATGCAGCGAGCTTCTT-
CAGCACGCATCCCGGTGTCCC-CG-GGGCTCAATC-CTGACCA-ATCA-CACACATCTGGTTGACC
Thelidium_toskalharjiense_43848   CATTATCGAG-
TTAGGGTCCTCCGGGCCCCAATCT-CCACCCT-TTGCCTACC-----ATGTTGCCCC--CGG---
CCTCTGCGCTGGGGCGACTTTAAAAAAATTT-------------------------C--TTTTTAAA-ATC-
TTCAACCATGTCGTCCCACGGGAGAAT-ATCAATCAATC-
AAAACTTTCAACAACGGATCTCTTGGTTCTGGCATCGATGAAGAACGCAGCGAAATGCGATAAGTAATGCGAA
TTGCAGAATTCCGTGAGTCATCGAATCTTTGAACGCACATTGCGCCCTTTGGTATTCCGAAGGGCATGCCTGT
TCGAGCGTCATTATCAACCCTCAAGCCCGGCTTGCCGTTGGGTCTCCACCGTTGG-CGACGACGGTGGACC-
CGAAAGATAATGGCAGAGTC-TGCGAGA--
CCCTGGATGCAGCGAGCTTTCTACAGCACGCATCGTAAGGGTTCCCGCGGGCTCGGTC-TTAACCG-
ATCAAA--CCATCTGGTTGACC
Thelidium_toskalharjiense_43090   CATTATCGAG-
TTAGGGTCCTCCGGGCCCCAATCT-CCAACCCTTTGCCTACC-----ATGTTGCCCC--CGG---
CCTCTGCGCTGGGGCGACTTTAAAAAAATTT-------------------------C--TTTTTAAA-ATC-
TTCAACCATGTCGTCCCACGGGAGAAT-ATCAATCAATC-
AAAACTTTCAACAACGGATCTCTTGGTTCTGGCATCGATGAAGAACGCAGCGAAATGCGATAAGTAATGCGAA
TTGCAGAATTCCGTGAGTCATCGAATCTTTGAACGCACATTGCGCCCTTTGGTATTCCGAAGGGCATGCCTGT
TCGAGCGTCATTATCAACCCTCAAGCCCGGCTTGCCGTTGGGTCTCCACCGTTGG-CGACGACGGTGGACC-
CGAAAGATAATGGCAGAGTC-TGCGAGA--
CCCTGGATGCAGCGAGCTTTCTACAGCACGCATCGTAAGGGTTCCCGCGGGCTCGGTC-TTAACCG-
ATCAAA--CCATCTGGTTGACC
Thelidium_toskalharjiense_43861   CATTATCGAG-
TTAGGGTCCTCCGGGCCCCAATCT-CCAACCCTTTGCCTACC-----ATGTTGCCCC--CGG---
CCTCTGCGCTGGGGCGACTTTAAAAAAATTT-------------------------C--TTTTTAAA-ATC-
TTCAACCATGTCGTCCCACGGGAGAAT-ATCAATCAATC-
AAAACTTTCAACAACGGATCTCTTGGTTCTGGCATCGATGAAGAACGCAGCGAAATGCGATAAGTAATGCGAA
TTGCAGAATTCCGTGAGTCATCGAATCTTTGAACGCACATTGCGCCCTTTGGTATTCCGAAGGGCATGCCTGT
TCGAGCGTCATTATCAACCCTCAAGCCCGGCTTGCCGTTGGGTCTCCACCGTTGG-CGACGACGGTGGACC-
CGAAAGATAATGGCAGAGTC-TGCGAGA--
CCCTGGATGCAGCGAGCTTTCTACAGCACGCATCGTAAGGGTTCCCGCGGGCTCGGTCNNNNNNNNNNNNNNN
NNNNNNNNNNNNNNNN
Thelidium_toskalharjiense_43211   CATTATCGAG-
TTAGGGTCCTCCGGGCCCCAATCT-CCAACCCTTTGCCTACC-----ATGTTGCCCC--CGG---
CCTCTGCGCTGGGGCGACTTTAAAAAAATTT-------------------------C--TTTTTAAA-ATC-

TTCAACCATGTCGTCCCACGGGAGAAT-ATCAATCAATC-
AAAACTTTCAACAACGGATCTCTTGGTTCTGGCATCGATGAAGAACGCAGCGAAATGCGATAAGTAATGCGAA
TTGCAGAATTCCGTGAGTCATCGAATCTTTGAACGCACATTGCGCCCTTTGGTATTCCGAAGGGCATGCCTGT
TCGAGCGTCATTATCAACCCTCAAGCCCGGCTTGCCGTTGGGTCTCCACCGTTGG-CGACGACGGTGGACC-
CGAAAGATAATGGCAGAGTC-TGCGAGA--
CCCTGGATGCAGCGAGCTTTCTACAGCACGCATCGTAAGGGTTCCCGCGGGCTCGGTC-TTAACCG-
ATCAAA--CCATCTGGTTGACC
Thelidium_toskalharjiense_43364 CATTATCGAG-
TTAGGGTCCTCCGGGCCCCAATCT-CCAACCCTTTGCCTACC-----ATGTTGCCCC--CGG---
CCTCTGCGCTGGGGCGACTTTAAAAAAATTT--------------------------C--TTTTTAAA-ATC-
TTCAACCATGTCGTCCCACGGGAGAAT-ATCAATCAATC-
AAAACTTTCAACAACGGATCTCTTGGTTCTGGCATCGATGAAGAACGCAGCGAAATGCGATAAGTAATGCGAA
TTGCAGAATTCCGTGAGTCATCGAATCTTTGAACGCACATTGCGCCCTTTGGTATTCCGAAGGGCATGCCTGT
TCGAGCGTCATTATCAACCCTCAAGCCCGGCTTGCCGTTGGGTCTCCACCGTTGG-CGACGACGGTGGACC-
CGAAAGATAATGGCAGAGTC-TGCGAGA--
CCCTGGATGCAGCGAGCTTTCTACAGCACGCATCGTAAGGGTTCCCGCGGGCTCGGTC-TTAACCG-
ATCAAA--CCATCTGGTTGACC
Thelidium_toskalharjiense_43398 CATTATCGAG-
TTAGGGTCCTCCGGGCCCCAATCT-CCAACCCTTTGCCTACC-----ATGTTGCCCC--CGG---
CCTCTGCGCTGGGGCGACTTTAAAAAAATTT--------------------------C--TTTTTAAA-ATC-
TTCAACCATGTCGTCCCACGGGAGAAT-ATCAATCAATC-
AAAACTTTCAACAACGGATCTCTTGGTTCTGGCATCGATGAAGAACGCAGCGAAATGCGATAAGTAATGCGAA
TTGCAGAATTCCGTGAGTCATCGAATCTTTGAACGCACATTGCGCCCTTTGGTATTCCGAAGGGCATGCCTGT
TCGAGCGTCATTATCAACCCTCAAGCCCGGCTTGCCGTTGGGTCTCCACCGTTGG-CGACGACGGTGGACC-
CGAAAGATAATGGCAGAGTC-TGCGAGA--
CCCTGGATGCAGCGAGCTTTCTACAGCACGCATCGTAAGGGTTCCCGCGGGCTCGGTC-TTAACCG-
ATCAAA--CCATCTGGTTGACC
Verrucaria_aethiobola_MT127203 CATTATCGAG-TTAAGGTCCTCTGGGCCCC-
ATCT-CCAACCCTTTGTCTACC-----ACGTCG-CTC--CGG---CGGGTATGCCGGAGTGATTT--
TAAAAATTT--------------------------C--TTTTTAAA-CTC-
TTTAACCATGACGTCCTCCGGGAGAAT-ATCAATGAATC-
AAAACTTTCAACAACGGATCTCTTGGTTCTGGCATCGATGAAGAACGCAGCGAAATGCGATAAGTAATGCGAA
TTGCAGAATTCCGTGAGTCATCGAATCTTTGAACGCACATTGCGCCCTTTGGTATTCCAAAGGGCATGCCTGT
TCGAGCGTCATTATCAACCCTCAAGCCCGGCTTGCAGCTGGATCC-CACCGTCGG-CGACGGCGGTGGATC-
TGAAAGATAATGGCAGAGTC-TGCGGGA--CCCTGGATGCAGCGAGCTTCTT-
TGGCACGCATCCCAACGGCCT-CGCGGGCTCGGTC-TGAACC--ATCACA-CTCATCTGGTTGACC
Verrucaria_latebrosa_FJ664864 CATTATCGAG-TCAGGGTCCTATGGGCCCC-
ATCT-CCAACCC-TTGCCTACT-----GCGATG-CCC--CGG---CATC-GTGCCGGGGCAGCGTT-
AAAAAATTT--------------------------C--TTTTTAA--TTC-
TTTAACCATGATGTCCTGCGGGAGAAG-ATCAATCAATT-
AAAACTTTCAACAACGGATCTCTTGGTTCTGGCATCGATGAAGAACGCAGCGAAATGCGATAAGTAATGCGAA
TTGCAGAATTCCGTGAGTCATCGAATCTTTGAACGCACATTGCGCCCTTTGGTATTCCGAAGGGCATGCCTGT
TCGAGCGTCATTATCAACCCTCAAGCCCGGCTTGCCGTTGGATCC-CACCGTTGG-CGACGGCGGTGGATC-
CGAAAGATAATGGCAGAGTC-TGCGAGA--CCCTGGATGCAGCGAGCTTC-T-
CAGCACGCATCTGAACGGCCC-CG-CGGCTCGGTC-TGAACCCGCCCA---TTCATCTGGTTGACC
Verrucaria_tephromela_MT127212 CATTATCGAG-TTAGGGTCCTCTGGGCCCC-
ATCT-CCAACCC-TTGTCTACT-----ATGTTG-CCC--CGG---CCTTTCCGCCGGGGTGACTTT-
TAAAAATTT--------------------------C--TTTTTAAA-TTC-
TTCAACCATGACGTCCTACGGGAAAAG-ATGAATCAATT-
AAAACTTTCAACAACGGATCTCTTGGTTCTGGCATCGATGAAGAACGCAGCGAAATGCGATAAGTAATGCGAA
TTGCAGAATTCCGTGAGTCATCGAATCTTTGAACGCACATTGCGCCCTTTGGTATTCCGAAGGGCATGCCTGT
TCGAGCGTCATTATCAACCCTCAAGCCCGGCTTGCTGTTGGATCT-CGCCGCCGG-CGACGGCGGTGGACC-
CGAAAGATAATGGCAGAGTC-TGCGGGA--CCCTGGATGCAGCGAGCTTC-T-
CAGCACGCATCTCAACGGCCC-CG-CGGCTCGGTC-TTAACCATACTCAA--TTATCTGGTTGACC
Thelidium_mendax_40152 CATTATCGAGTCCGGGGTCCTCTGGGCCCC-
TTCTCCCAACCC-TTGTCTACC-----ACGTTG-CCC--TGG---CGTCCGCGCCGGGACAGCTT--
AAAAAATTT--------------------------C-
TTTTTTAAACCCCTTTTAACCATGACGTCCTACGGGAGAAG-ATCAATCAATC-

AAAACTTTCAACAACGGATCTCTTGGTTCTGGCATCGATGAAGAACGCAGCGAAATGCGATAAGTAATGCGAA
TTGCAGAATTCCGTGAGTCATCGAATCTTTGAACGCACATTGCGCCCTTTGGTATTCCGAAGGGCATGCCTGT
TCGAGCGTCATTATCAACCCTCAAGCCCGGCTTGCTGTTGGATCC-CATCGCGGT-CGCCCGCGGTGGATC-
CGAAAGATAATGGCAGAGTCTTGCGAGA--CTTTGGATGCAGCGAGCTTCTT-
CAGCACGCATCTGAACCGCCT-CG-CGGCTCGGTCTAAAACCA-ATCAAT-TTCATCTGGTTGACC
Thelidium_mendax_39179 CATTATCGAGTCCGGGGTCCTCTGGGCCCC-
TTCTCCCAACCC-TTGTCTACC-----ACGTTG-CCC--TGG---CGTCCGCGCCGGGACAGCTT--
AAAAAATTT-------------------------C-
TTTTTTAAACCCCTTTTAACCATGACGTCCTACGGGAGAAG-ATCAATCAATC-
AAAACTTTCAACAACGGATCTCTTGGTTCTGGCATCGATGAAGAACGCAGCGAAATGCGATAAGTAATGCGAA
TTGCAGAATTCCGTGAGTCATCGAATCTTTGAACGCACATTGCGCCCTTTGGTATTCCGAAGGGCATGCCTGT
TCGAGCGTCATTATCAACCCTCAAGCCCGGCTTGCTGTTGGATCC-CATCGCGGT-CGCCCGCGGTGGATC-
CGAAAGATAATGGCAGAGTCTTGCGAGA--CTTTGGATGCAGCGAGCTTCTT-
CAGCACGCATCTGAACCGCCT-CG-CGGCTCGGTCTAAAACCA-ATCAAT-TTCATCTGGTTGACC
Thelidium_mendax_42503 CATTATCGAGTCCGGGGTCCTCTGGGCCCC-
TTCTCCCAACCC-TTGTCTACC-----ACGTTG-CCC--TGG---CGTCCGCGCCGGGACAGCTT--
AAAAAATTT-------------------------C-
TTTTTTAAACCCCTTTTAACCATGACGTCCTACGGGAGAAG-ATCAATCAATC-
AAAACTTTCAACAACGGATCTCTTGGTTCTGGCATCGATGAAGAACGCAGCGAAATGCGATAAGTAATGCGAA
TTGCAGAATTCCGTGAGTCATCGAATCTTTGAACGCACATTGCGCCCTTTGGTATTCCGAAGGGCATGCCTGT
TCGAGCGTCATTATCAACCCTCAAGCCCGGCTTGCTGTTGGATCC-CATCGCGGT-CGCCCGCGGTGGATC-
CGAAAGATAATGGCAGAGTCTTGCGAGA--CTTTGGATGCAGCGAGCTTCTT-
CAGCACGCATCTGAACCGCCT-CG-CGGCTCGGTCTAAAACCA-ATCAAT-TTCATCNNNNNNNNNN
Thelidium_mendax_42502 CATTATCGAGTCCGGGGTCCTCTGGGCCCC-
TTCTCCCAACCC-TTGTCTACC-----ACGTTG-CCC--TGG---CGTCCGCGCCGGGACAGCTT--
AAAAAATTT-------------------------C-
TTTTTTAAACCCCTTTTAACCATGACGTCCTACGGGAGAAG-ATCAATCAATC-
AAAACTTTCAACAACGGATCTCTTGGTTCTGGCATCGATGAAGAACGCAGCGAAATGCGATAAGTAATGCGAA
TTGCAGAATTCCGTGAGTCATCGAATCTTTGAACGCACATTGCGCCCTTTGGTATTCCGAAGGGCATGCCTGT
TCGAGCGTCATTATCAACCCTCAAGCCCGGCTTGCTGTTGGATCC-CATCGCGGT-CGCCCGCGGTGGATC-
CGAAAGATAATGGCAGAGTCTTGCGAGA--CTTTGGATGCAGCGAGCTTCTT-
CAGCACGCATCTGAACCGCCT-CG-CGGCTCGGTCTAAAACCA-ATCAANNNNNNNNNNNNNNNNN
Thelidium_declivum_40037 CATTATCGAGTCCGGGGTCCTCTGGGCCCC-
GTCT-CCAACCC-TTGCTTACC-----ACGTTG-CTC--TGG---CGTCCGCGCCCGGACAGCTT--
AAAAAATTT-------------------------
CTTTTTTTAAACCCCTTTTAACCATGACGTCCTACGGGAGAAG-ATCAATCAATC-
AAAACTTTCAACAACGGATCTCTTGGTTCTGGCATCGATGAAGAACGCAGCGAAATGCGATAAGTAATGCGAA
TTGCAGAATTCCGTGAGTCATCGAATCTTTGAACGCACATTGCGCCCTTTGGTATTCCGAAGGGCATGCCTGT
TCGAGCGTCATTATCAACCCTCAAGCCCGGCTTGCTGTTGGGTCC-CACCGCGGT-CACCCGCGGTGGATC-
CGAAAGATAATGGCAGAGTC-TGCGGGA--CTTTGGATGCAGCGAGCTTCTT-
CAGCACGCATCTGAACCGTCT-CG-CGGCTCGGTC-TCAACCA-ATCAAT-TTCATCTGGTTGACC
Thelidium_declivum_44554 CATTATCGAGTCCGGGGTCCTCTGGGCCCC-
GTCT-CCAACCC-TTGCTTACC-----ACGTTG-CTC--TGG---CGTCCGCGCCCGGACAGCTT--
AAAAAATTT-------------------------
CTTTTTTTAAACCCCTTTTAACCATGACGTCCTACGGGAGAAG-ATCAATCAATC-
AAAACTTTCAACAACGGATCTCTTGGTTCTGGCATCGATGAAGAACGCAGCGAAATGCGATAAGTAATGCGAA
TTGCAGAATTCCGTGAGTCATCGAATCTTTGAACGCACATTGCGCCCTTTGGTATTCCGAAGGGCATGCCTGT
TCGAGCGTCATTATCAACCCTCAAGCCCGGCTTGCTGTTGGGTCC-CACCGCGGT-CACCCGCGGTGGATC-
CGAAAGATAATGGCAGAGTC-TGCGGGA--CTTTGGATGCAGCGAGCTTCTT-
CAGCACGCATCTGAACCGTCT-CG-CGGCTCGGTC-TCAACCA-ATCAAT-TTCATCTGGTTGACC
Thelidium_declivum_36334 CATTATCGAGTCCGGGGTCCTCTGGGCCCC-
GTCT-CCAACCC-TTGCTTACC-----ACGTTG-CTC--TGG---CGTCCGCGCCCGGACAGCTT--
AAAAAATTT-------------------------
CTTTTTTTAAACCCCTTTTAACCATGACGTCCTACGGGAGAAG-ATCAATCAATC-
AAAACTTTCAACAACGGATCTCTTGGTTCTGGCATCGATGAAGAACGCAGCGAAATGCGATAAGTAATGCGAA
TTGCAGAATTCCGTGAGTCATCGAATCTTTGAACGCACATTGCGCCCTTTGGTATTCCGAAGGGCATGCCTGT
TCGAGCGTCATTATCAACCCTCAAGCCCGGCTTGCTGTTGGGTCC-CACCGCGGT-CACCCGCGGTGGATC-

CGAAAGATAATGGCAGAGTC-TGCGGGA--CTTTGGATGCAGCGAGCTTCTT-
CAGCACGCATCTGAACCGTCT-CG-CGGCTCGGTC-TCAACCA-ATCAAT-TTCATCTGGTTGACC
Thelidium_declivum_39640 CATTATCGAGTCCGGGGTCCTCTGGGCCCC-
GTCT-CCAACCC-TTGCTTACC-----ACGTTG-CTC--TGG---CGTCCGCGCCCGGACAGCTT--
AAAAAATTT--------------------------
CTTTTTTTAAACCCCTTTTAACCATGACGTCCTACGGGAGAAG-ATCAATCAATC-
AAAACTTTCAACAACGGATCTCTTGGTTCTGGCATCGATGAAGAACGCAGCGAAATGCGATAAGTAATGCGAA
TTGCAGAATTCCGTGAGTCATCGAATCTTTGAACGCACATTGCGCCCTTTGGTATTCCGAAGGGCATGCCTGT
TCGAGCGTCATTATCAACCCTCAAGCCCGGCTTGCTGTTGGGTCC-CACCGCGGT-CACCCGCGGTGGATC-
CGAAAGATAATGGCAGAGTC-TGCGGGA--CTTTGGATGCAGCGAGCTTCTT-
CAGCACGCATCTGAACCGTCT-CG-CGGCTCGGTC-TCAACCA-ATCAAT-TTCATCTGGTTGACC
Thelidium_declivum_45123 CATTATCGAGTCCGGGGTCCTCTGGGCCCC-
GTCT-CCAACCC-TTGCTTACC-----ACGTTG-CTC--TGG---CGTCCGCGCCCGGACAGCTT--
AAAAAATTT--------------------------
CTTTTTTTAAACCCCTTTTAACCATGACGTCCTACGGGAGAAG-ATCAATCAATC-
AAAACTTTCAACAACGGATCTCTTGGTTCTGGCATCGATGAAGAACGCAGCGAAATGCGATAAGTAATGCGAA
TTGCAGAATTCCGTGAGTCATCGAATCTTTGAACGCACATTGCGCCCTTTGGTATTCCGAAGGGCATGCCTGT
TCGAGCGTCATTATCAACCCTCAAGCCCGGCTTGCTGTTGGGTCC-CACCGCGGT-CACCCGCGGTGGATC-
CGAAAGATAATGGCAGAGTC-TGCGGGA--CTTTGGATGCAGCGAGCTTCTT-
CAGCACGCATCTGAACCGTCT-CG-CGGCTCGGTC-TCAACCA-ATCAAT-TTCATCTGGTTGACC
Thelidium_declivum_40047 CATTATCGAGTCCGGGGTCCTCTGGGCCCC-
GTCT-CCAACCC-TTGCTTACC-----ACGTTG-CTC--TGG---CGTCCGCGCCCGGACAGCTT--
AAAAAATTT--------------------------
CTTTTTTTAAACCCCTTTTAACCATGACGTCCTACGGGAGAAG-ATCAATCAATC-
AAAACTTTCAACAACGGATCTCTTGGTTCTGGCATCGATGAAGAACGCAGCGAAATGCGATAAGTAATGCGAA
TTGCAGAATTCCGTGAGTCATCGAATCTTTGAACGCACATTGCGCCCTTTGGTATTCCGAAGGGCATGCCTGT
TCGAGCGTCATTATCAACCCTCAAGCCCGGCTTGCTGTTGGGTCC-CACCGCGGT-CACCCGCGGTGGATC-
CGAAAGATAATGGCAGAGTC-TGCGGGA--CTTTGGATGCAGCGAGCTTCTT-
CAGCACGCATCTGAACCGTCT-CG-CGGCTCGGTC-TCAACCA-ATCAAT-TTCATCTGGTTGACC
Thelidium_declivum_39997 CATTATCGAGTCCGGGGTCCTCTGGGCCCC-
GTCT-CCAACCC-TTGCTTACC-----ACGTTG-CTC--TGG---CGTCCGCGCCCGGACAGCTT--
AAAAAATTT--------------------------
CTTTTTTTAAACCCCTTTTAACCATGACGTCCTACGGGAGAAG-ATCAATCAATC-
AAAACTTTCAACAACGGATCTCTTGGTTCTGGCATCGATGAAGAACGCAGCGAAATGCGATAAGTAATGCGAA
TTGCAGAATTCCGTGAGTCATCGAATCTTTGAACGCACATTGCGCCCTTTGGTATTCCGAAGGGCATGCCTGT
TCGAGCGTCATTATCAACCCTCAAGCCCGGCTTGCTGTTGGGTCC-CACCGCGGT-CACCCGCGGTGGATC-
CGAAAGATAATGGCAGAGTC-TGCGGGA--CTTTGGATGCAGCGAGCTTCTT-
CAGCACGCATCTGAACCGTCT-CG-CGGCTCGGTC-TCAACCA-ATCAAT-TTCATCTGGTTGACC
Thelidium_declivum_39780b CATTATCGAGTCCGGGGTCCTCTGGGCCCC-
GTCT-CCAACCC-TTGCTTACC-----ACGTTG-CTC--TGG---CGTCCGCGCCCGGACAGCTT--
AAAAAATTT--------------------------
CTTTTTTTAAACCCCTTTTAACCATGACGTCCTACGGGAGAAG-ATCAATCAATC-
AAAACTTTCAACAACGGATCTCTTGGTTCTGGCATCGATGAAGAACGCAGCGAAATGCGATAAGTAATGCGAA
TTGCAGAATTCCGTGAGTCATCGAATCTTTGAACGCACATTGCGCCCTTTGGTATTCCGAAGGGCATGCCTGT
TCGAGCGTCATTATCAACCCTCAAGCCCGGCTTGCTGTTGGGTCC-CACCGCGGT-CACCCGCGGTGGATC-
CGAAAGATAATGGCAGAGTC-TGCGGGA--CTTTGGATGCAGCGAGCTTCTT-
CAGCACGCATCTGAACCGTCT-CG-CGGCTCGGTC-TCAACCA-ATCAAT-TTCATCTGGTTGACC
Thelidium_declivum_35996 NNNNNTCGAGTCCGGGGTCCTCTGGGCCCC-
GTCT-CCAACCC-TTGCTTACC-----ACGTTG-CTC--TGG---CGTCCGCGCCCGGACAGCTT--
AAAAAATTT--------------------------
CTTTTTTTAAACCCCTTTTAACCATGACGTCCTACGGGAGAAG-ATCAATCAATC-
AAAACTTTCAACAACGGATCTCTTGGTTCTGGCATCGATGAAGAACGCAGCGAAATGCGATAAGTAATGCGAA
TTGCAGAATTCCGTGAGTCATCGAATCTTTGAACGCACATTGCGCCCTTTGGTATTCCGAAGGGCATGCCTGT
TCGAGCGTCATTATCAACCCTCAAGCCCGGCTTGCTGTTGGGTCC-CACCGCGGT-CACCCGCGGTGGATC-
CGAAAGATAATGGCAGAGTC-TGCGGGA--CTTTGGATGCAGCGAGCTTCTT-
CAGCACGCATCTGAACCGTCT-CG-CGGCTCGGTC-TCAACCA-ATCAAT-TTCATCTGGTTGACC
Thelidium_huuskonenii_43243 CATTATCGAG-TTAGGGTCTTCTGGGCCCC-
ATCT-CTAACCC-TTGCCCACC-----ACGTTG-CCC--CGG---

CATTCGTGCCGGGGAAACTTTTGAAAAATTT--------------------------C-TTTTTGAAA-CCC-
TTCAACCATGACGTCCCACGGGAAAAT-ATCAATCAATC-
AAAACTTTCAACAACGGATCTCTTGGTTCTGGCATCGATGAAGAACGCAGCGAAATGCGATAAGTAATGCGAA
TTGCAGAATTCCGTGAGTCATCGAATCTTTGAACGCACATTGCGCCCTTTGGTATTCCGAAGGGCATGCCTGT
TCGAGCGTCATTATCAACCCTCAAGCCCGGCTTGCCGTTGGATCC-CACCGCTGG-CGACGGCGGTGGATC-
CGAAAGATAATGGCAGAGTC-TGCGGGA--CCCTGGATGCAGCGAGCTTCTT-
CAGCACGCATCTCGGTGGCCT-CG-CGGCTCGGTC-TTAACCC-ATCACA-TTTATCTGGTTGACC
Thelidium_huuskonenii_44167 CATTATCGAG-TTAGGGTCTTCTGGGCCCC-
ATCT-CTAACCC-TTGCCCACC-----ACGTTG-CCC--CGG---
CATTCGTGCCGGGGAAACTTTTGAAAAATTT--------------------------C-TTTTTGAAA-CCC-
TTCAACCATGACGTCCCACGGGAAAAT-ATCAATCAATC-
AAAACTTTCAACAACGGATCTCTTGGTTCTGGCATCGATGAAGAACGCAGCGAAATGCGATAAGTAATGCGAA
TTGCAGAATTCCGTGAGTCATCGAATCTTTGAACGCACATTGCGCCCTTTGGTATTCCGAAGGGCATGCCTGT
TCGAGCGTCATTATCAACCCTCAAGCCCGGCTTGCCGTTGGATCC-CACCGCTGG-CGACGGCGGTGGATC-
CGAAAGATAATGGCAGAGTC-TGCGGGA--CCCTGGATGCAGCGAGCTTCTT-
CAGCACGCATCTCGGTGGCCT-CG-CGGCTCGGTC-TTAACCC-ATCACA-TTTATCTGGTTGACC
Thelidium_huuskonenii_43246 CATTATCGAG-TTAGGGTCTTCTGGGCCCC-
ATCT-CTAACCC-TTGCCCACC-----ACGTTG-CCC--CGG---
CATTCGTGCCGGGGAAACTTTTGAAAAATTT--------------------------C-TTTTTGAAA-CCC-
TTCAACCATGACGTCCCACGGGAAAAT-ATCAATCAATC-
AAAACTTTCAACAACGGATCTCTTGGTTCTGGCATCGATGAAGAACGCAGCGAAATGCGATAAGTAATGCGAA
TTGCAGAATTCCGTGAGTCATCGAATCTTTGAACGCACATTGCGCCCTTTGGTATTCCGAAGGGCATGCCTGT
TCGAGCGTCATTATCAACCCTCAAGCCCGGCTTGCCGTTGGATCC-CACCGCTGG-CGACGGCGGTGGATC-
CGAAAGATAATGGCAGAGTC-TGCGGGA--CCCTGGATGCAGCGAGCTTCTT-
CAGCACGCATCTCGGTGGCCT-CG-CGGCTCGGTC-TTAACCC-ATCACA-TTTATCTGGTTGACC
Thelidium_huuskonenii_31576 CATTATCGAG-TTAGGGTCTTCTGGGCCCC-
ATCT-CTAACCC-TTGCCCACC-----ACGTTG-CCC--CGG---
CATTCGTGCCGGGGAAACTTTTGAAAAATTT--------------------------C-TTTTTGAAA-CCC-
TTCAACCATGACGTCCCACGGGAAAAT-ATCAATCAATC-
AAAACTTTCAACAACGGATCTCTTGGTTCTGGCATCGATGAAGAACGCAGCGAAATGCGATAAGTAATGCGAA
TTGCAGAATTCCGTGAGTCATCGAATCTTTGAACGCACATTGCGCCCTTTGGTATTCCGAAGGGCATGCCTGT
TCGAGCGTCATTATCAACCCTCAAGCCCGGCTTGCCGTTGGATCC-CACCGCTGG-CGACGGCGGTGGATC-
CGAAAGATAATGGCAGAGTC-TGCGGGA--CCCTGGATGCAGCGAGCTTCTT-
CAGCACGCATCTCGGTGGCCT-CG-CGGCTCGGTC-TTAACCC-ATCACA-TTTATCTGGTTNNNN
Thelidium_pertusatii_EU249471 CATTATCGAG-TAAGGGTCCTCTGGGCCCC-
ATCT-CCAACCC-TTGTCTACC-----ACATGC-CTC--CGG---CCTCTCCACCGGGGTCACTTT----
AAATTT--------------------------C--TTTTTAAA-TTC-
TTCAACCATGACGTCCTACGGGAGAAA-ATCAATTAACT-
AAAACTTTCAACAACGGATCTCTTGGTTCTGGCATCGATGAAGAACGCAGCGAAATGCGATAAGTAATGCGAA
TTGCAGAATTCCGTGAGTCATCGAATCTTTGAACGCACATTGCGCCCTTTGGTATTCCGAAGGGCATGCCTGT
TCGAGCGTCATTATCAACCCTCAAGCTCGGCTTGCTGTTGGGTCT-CACCGCCGA-
CAACGGCGGCGGATCTCAAAAGATGATGGCAGAGCC-TGCTTGA--CCCTGGATGCAGCGAGCTTCTT-
CAGCACGTATCCCGGTGGCCT-TG-TGGCTCAGTC-TTAACCA-AGTACA-TTAATCTGGTTGACC
Thelidium_pyrenophorum_EU553500 CATTATCGAG-TCAGGGTCCTCTGGGCCCC-
ATCT-CCAACCC-TTGTCTACC-----ACGTGG-CTC--CGG---CGTGACTGCCGGGGTCGCTT--
TGAAAATTT--------------------------C--TTTTTAAA-TTC-
TTCAACCATGACGTCCTGCGGGAGAAT-ATCAATCAATC-
AAAACTTTCAACAACGGATCTCTTGGTTCTGGCATCGATGAAGAACGCAGCGAAATGCGATAAGTAATGCGAA
TTGCAGAATTCCGTGAGTCATCGAATCTTTGAACGCACATTGCGCCCTTTGGTATTCCGAAGGGCATGCCTGT
TCGAGCGTCATTATCAACCCTCAAGCTCGGCTTGCTGTTGGATCC-CACCGTCGG-CAACGGCGGTGGATC-
CCAAAGATAATGGCAGAGTC-TGCTGGA--CCCTGGATGCAGCGAGCTTCTT-
CAGCACGCATCCCAGCGGCCT-CG-TGGCTCCGTC-TTGACCNNNNNNNNNNNNNNNNNNNNNNN
Thelidium_sp1_43208 CATTATCGAG-TTCGGGTCTTCTGGGCCCC-
ATCT-CCAACCT-TTGTCTACC-----ACGTGG-CTC--CGG---CGTCCCTGCCGGGGTCACTT--
TGAAAATTT--------------------------C--TTTTTAAA-TTC-
TTCAACCATGACGTCCTACGGGAGAAT-ATCAATAAATG-
AAAACTTTCAACAACGGATCTCTTGGTTCTGGCATCGATGAAGAACGCAGCGAAATGCGATAAGTAATGCGAA
TTGCAGAATTCCGTGAGTCATCGAATCTTTGAACGCACATTGCGCCCTTTGGTATTCCGAAGGGCATGCCTGT

TCGAGCGTCATTATCAACCCTCAAGCTCGGCTTGCTGTTGGATCT-CGCCGCCGA-CCACGGCGGCGGATC-
CCAAAGACAGTGGCAGAGTC-TGCTTGA--CCCTGGATGCAGCGAGCTTCTT-
TAGCACGCATCCCAGTGGCCT-TG-GGGCTCAGTC-TGAACCA-ATCACA-TTTATCTGGTTGACC
Thelidium_methorium_FJ645267   CATTATCGAG-TTAGGGTCCTCTGGGCCCC-
ATCT-CCAACCC-TTGCCTATT-----ACGCGG-CTC--CGG---CGTCTCTGCCGGGGTCACTT--
TGAAAATTT--------------------------C--TTTTTAAA-TTC-
TTCAACCATGACGTCCCACGGGAGAAT-ATCAATTGATT-
AAAACTTTCAACAACGGATCTCTTGGTTCTGGCATCGATGAAGAACGCAGCGAAATGCGATAAGTAATGCGAA
TTGCAGAATTCCGTGAGTCATCGAATCTTTGAACGCACATTGCGCCCTTTGGTATTCCGAAGGGCATGCCTGT
TCGAGCGTCATTATCAACCCTCAAGCTCGGCTTGCTGTTGGATCT-CACCGTCGA-CCACGGCGGTGAATC-
TCAAAGATAATGGCAGAGTC-TGCTTGA--CCCTGGATGCAGCGAGCTTCTT-
CAGCACGCATCCCGGCGGCCT-TG-TGGCTCAGTC-TTAACCA-ATCACA-TTTATCTNNNNNNNN
Thelidium_methorium_MT127220   CATTATCGAG-TTAGGGTTCTCTGGGCCCC-
ATCT-CCAACCC-TTGCCTATT-----ACGCGG-CTC--CGG---CGTCTCTGCCGGGGTCACTT--
TGAAAATTT--------------------------C--TTTTTAAA-TTC-
TTCAACCATGACGTCCTACGGGAGAAT-ATCAATTGATT-
AAAACTTTCAACAACGGATCTCTTGGTTCTGGCATCGATGAAGAACGCAGCGAAATGCGATAAGTAATGCGAA
TTGCAGAATTCCGTGAGTCATCGAATCTTTGAACGCACATTGCGCCCTTTGGTATTCCGAAGGGCATGCCTGT
TCGAGCGTCATTATCAACCCTCAAGCTCGGCTTGCTGTTGGATCT-CACCGCCGA-CCACGGCGGCGAATC-
TCAAAGATAATGGCAGAGTC-TGCTTGA--CCCCGGATGCAGCGAGCTTCTT-
CAGCACGCATCCCGGCGGCCT-TG-TGGCTCAGTC-TTAACCA-ATCACA-TTTATCTGGTGANNN
Thelidium_sallaense_44902   CATTATCGAG-TTAGGGTCTTCTGGGCCCC-
ATCT-CCAACCC-TTGTCTACC-----ACGCGG-CTC--CGG---CGTCTCTGCCGGGGTCACTT--
TAAAAATTT--------------------------C--TTTTTAAA-TTC-
TTCAACCATGACGTCCTACGGGAGAAT-ATTAATGAATT-
AAAACTTTCAACAACGGATCTCTTGGTTCTGGCATCGATGAAGAACGCAGCGAAATGCGATAAGTAATGCGAA
TTGCAGAATTCCGTGAGTCATCGAATCTTTGAACGCACATTGCGCCCTTTGGTATTCCGAAGGGCATGCCTGT
TCGAGCGTCATTATCACCCCTCAAGCTCGGCTTGCTGTTGGATCT-CACCGCCGA-CAACGGCGGCGGATC-
TCAAAGATAATGGCAGAGTC-TGCTTGA--CCCTGGATGCAGCGAGCTTCTT-
CAGCACGCATCCCAGCGGCCT-TG-TGGCTCGGTC-TTAACCA-ATCACA-TACATCTGGTTGACC
Thelidium_pseudoauruntii_45374   CATTATCGAG-TCAGGGTCTTCTGGGCCCC-
ATCT-CCAACCC-TTGTCTACC-----ACGCGG-CTC--CGG---CGTCTCTGCCAGGGTCGCTT--
TAAAAATTT--------------------------C--TTTTTAAA-TTC-
TTCAACCCTGACGTCCTACGGGAGAAT-ATTAATGAATT-
AAAACTTTCAACAACGGATCTCTTGGTTCTGGCATCGATGAAGAACGCAGCGAAATGCGATAAGTAATGCGAA
TTGCAGAATTCCGTGAGTCATCGAATCTTTGAACGCACATTGCGCCCTTTGGTATTCCAAAGGGCATGCCTGT
CCGAGCGTCATTATCAACCCTCAAGCTCGGCTTGCTGTTGGATCT-CACCGCCGA-CAACGGCGGCGGATC-
TCAAAGATAATGGCAGAGTC-TGCCGGA--CCCTGGATGCAGCGAGCTTCTT-
CAGCACGTATCCCAGTGGCCT-TG-TGGCTCAGTC-TTAACCA-ATTACA-TTTATCTGGTTGACC
Thelidium_pseudoauruntii_45371   CATTATCGAG-TCAGGGTCTTCTGGGCCCC-
ATCT-CCAACCC-TTGTCTACC-----ACGCGG-CTC--CGG---CGTCTCTGCCAGGGTCGCTT--
TAAAAATTT--------------------------C--TTTTTAAA-TTC-
TTCAACCCTGACGTCCTACGGGAGAAT-ATTAATGAATT-
AAAACTTTCAACAACGGATCTCTTGGTTCTGGCATCGATGAAGAACGCAGCGAAATGCGATAAGTAATGCGAA
TTGCAGAATTCCGTGAGTCATCGAATCTTTGAACGCACATTGCGCCCTTTGGTATTCCAAAGGGCATGCCTGT
CCGAGCGTCATTATCAACCCTCAAGCTCGGCTTGCTGTTGGATCT-CACCGCCGA-CAACGGCGGCGGATC-
TCAAAGATAATGGCAGAGTC-TGCCGGA--CCCTGGATGCAGCGAGCTTCTT-
CAGCACGTATCCCAGTGGCCT-TG-TGGCTCAGTC-TTAACCA-ATTACA-TTTATCTGGTTGACC
Thelidium_auruntii_434446   CATTATCGAG-TTAGGGTCTGCTGGGCCCC-
ATCT-TCAACCC-TTGTCTACC-----ACGCGG-CTC--CGG---CGTCTCTGCCGGGGTCACTT--
TAAAAATTT--------------------------C--TTTTTAAA-TTC-
TTCAACCATGACGTCCCACGGGAGAAT-ATTAATTAATT-
AAAACTTTCAACGACGGATCTCTTGGTTCTGGCATCGATGAAGAACGCAGCGAAATGCGATAAGTAATGCGAA
TTGCAGAATTCCGTGAGTCATCGAATCTTTGAACGCACATTGCGCCCTTTGGTATTCCGAAGGGCATGCCTGT
CCGAGCGTCATTATCAACCCTCAAGCTCGGCTTGCTGTTGGATCT-CACCGCCGG-CAACGGCGGCGGATC-
TCAAAGATAATGGCAGAGTC-TGCCGGA--CCCTGGATGCAGCGAGCTTCTT-
CAGCACGTATCCCAGTGGCCT-TG-TGGCTCAGTC-TTAACCA-ATTACG-TTTATC-GGTTGACC

Thelidium_auruntii_43470 CATTATCGAG-TTAGGGTCTGCTGGGCCCC-
ATCT-TCAACCC-TTGTCTACC-----ACGCGG-CTC--CGG---CGTCTCTGCCGGGGTCACTT--
TAAAAATTT-------------------------C--TTTTTAAA-TTC-
TTCAACCATGACGTCCCACGGGAGAAT-ATTAATTAATT-
AAAACTTTCAACAACGGATCTCTTGGTTCTGGCATCGATGAAGAACGCAGCGAAATGCGATAAGTAATGCGAA
TTGCAGAATTCCGTGAGTCATCGAATCTTTGAACGCACATTGCGCCCTTTGGTATTCCGAAGGGCATGCCTGT
CCGAGCGTCATTATCAACCCTCAAGCTCGGCTTGCTGTTGGATCT-CACCGCCGG-CAACGGCGGCGGATC-
TCAAAGATAATGGCAGAGTC-TGCCGGA--CCCTGGATGCAGCGAGCTTCTT-
CAGCACGTATCCCAGTGGCCT-TG-TGGCTCAGTC-TTAACCA-ATTACG-TTTATC-GGTTGACC
Thelidium_auruntii_43414 CATTATCGAG-TTAGGGTCTGCTGGGCCCC-
ATCT-TCAACCC-TTGTCTACC-----ACGCGG-CTC--CGG---CGTCTCTGCCGGGGTCACTT--
TAAAAATTT-------------------------C--TTTTTAAA-TTC-
TTCAACCATGACGTCCCACGGGAGAAT-ATTAATTAATT-
AAAACTTTCAACAACGGATCTCTTGGTTCTGGCATCGATGAAGAACGCAGCGAAATGCGATAAGTAATGCGAA
TTGCAGAATTCCGTGAGTCATCGAATCTTTGAACGCACATTGCGCCCTTTGGTATTCCGAAGGGCATGCCTGT
CCGAGCGTCATTATCAACCCTCAAGCTCGGCTTGCTGTTGGATCT-CACCGCCGG-CAACGGCGGCGGATC-
TCAAAGATAATGGCAGAGTC-TGCCGGA--CCCTGGATGCAGCGAGCTTCTT-
CAGCACGTATCCCAGTGGCCT-TG-TGGCTCAGTC-TTAACCA-ATTACG-TTTATC-GGTTGACC
Thelidium_auruntii_36339 CATTATCGAG-TTAGGGTNTGCTGGGCCCC-
ATCT-CCAACCC-TTGTCTACC-----ACGCGG-CTC--CGG---CGTCTCTGCCGGGGTCACTT--
TAAAAATTT-------------------------C--TTTTTAAA-TTC-
TTCAACCATGACGTCCCACGGGAGAAT-ATTAATTAATT-
AAAACTTTCAACAACGGATCTCTTGGTTCTGGCATCGATGAAGAACGCAGCGAAATGCGATAAGTAATGCGAA
TTGCAGAATTCCGTGAGTCATCGAATCTTTGAACGCACATTGCGCCCTTTGGTATTCCGAAGGGCATGCCTGT
CCGAGCGTCATTATCAACCCTCAAGCTCGGCTTGCTGTTGGATCT-CACCGCCGG-CAACGGCGGCGGATC-
TCAAAGATAATGGCAGAGTC-TGCCGGA--CCCTGGATGCAGCGAGCTTCTT-
CAGCACGTATCCCAGTGGCCT-TG-TGGCTCAGTC-TTAACCA-ATTACG-TTTATC-GGTTGACC
Thelidium_auruntii_43905 CATTATCGAG-TTAGGGTCTGCTGGGCCCC-
ATCT-CCAACCC-TTGTCTACC-----ACGCGG-CTC--CGG---CGTCTCTGCCGGGGTCACTT--
TAAAAATTT-------------------------C--TTTTTAAA-TTC-
TTCAACCATGACGTCCCACGGGAGAATATTTAATTAATT-
AAAACTTTCAACAACGGATCTCTTGGTTCTGGCATCGATGAAGAACGCAGCGAAATGCGATAAGTAATGCGAA
TTGCAGAATTCCGTGAGTCATCGAATCTTTGAACGCACATTGCGCCCTTTGGTATTCCGAAGGGCATGCCTGT
CCGAGCGTCATTATCAACCCTCAAGCTCGGCTTGCTGTTGGATCT-CACCGCCGG-CAACGGCGGCGGATC-
TCAAAGATAATGGCAGAGTC-TGCCGGA--CCCTGGATGCAGCGAGCTTCTT-
CAGCACGTATCCCAGTGGCCT-TG-TGGCTCAGTC-TTAACCA-ATTACG-TTTATC-GGTTGACC
Thelidium_auruntii_45171 CATTATCGAG-TTAGGGTCTGCTGGGCCCC-
ATCT-CCAACCC-TTGTCTACC-----ACGCGG-CTC--CGG---CGTCTCTGCCGGGGTCACTT--
TAAAAATTT-------------------------C--TTTTTAAA-TTC-
TTCAACCATGACGTCCCACGGGAGAAT-ATTAATTAATT-
AAAACTTTCAACAACGGATCTCTTGGTTCTGGCATCGATGAAGAACGCAGCGAAATGCGATAAGTAATGCGAA
TTGCAGAATTCCGTGAGTCATCGAATCTTTGAACGCACATTGCGCCCTTTGGTATTCCGAAGGGCATGCCTGT
CCGAGCGTCATTATCAACCCTCAAGCTCGGCTTGCTGTTGGATCT-CACCGCCGG-CAACGGCGGCGGATC-
TCAAAGATAATGGCAGAGTC-TGCCGGA--CCCTGGATGCAGCGAGCTTCTT-
CAGCACGTATCCCAGTGGCCT-TG-TGGCTCAGTC-TTAACCA-ATTACG-TTTATC-GGTTGACC
Thelidium_auruntii_43829 CATTATCGAG-TTAGGGTCTGCTGGGCCCC-
ATCT-CCAACCC-TTGTCTACC-----ACGCGG-CTC--CGG---CGTCTCTGCCGGGGTCACTT--
TAAAAATTT-------------------------C--TTTTTAAA-TTC-
TTCAACCATGACGTCCCACGGGAGAAT-ATTAATTAATT-
AAAACTTTCAACAACGGATCTCTTGGTTCTGGCATCGATGAAGAACGCAGCGAAATGCGATAAGTAATGCGAA
TTGCAGAATTCCGTGAGTCATCGAATCTTTGAACGCACATTGCGCCCTTTGGTATTCCGAAGGGCATGCCTGT
CCGAGCGTCATTATCAACCCTCAAGCTCGGCTTGCTGTTGGATCT-CACCGCCGG-CAACGGCGGCGGATC-
TCAAAGATAATGGCAGAGTC-TGCCGGA--CCCTGGATGCAGCGAGCTTCTT-
CAGCACGTATCCCAGTGGCCT-TG-TGGCTCAGTC-TTAACCA-ATTACG-TTTATC-GGTTGACC
Verrucaria_vacillans_MT229831 CATTATCGAGTTTAGGGTCCTCTGGGCCCC-
ATCT-CCAACCC-TTGTCTACC-----ACGTCG-CCC--CGG---CG-CTCAGCCAGGGTGGCTT--
AAAAAATTT-------------------------C--TTTTTAAA-TTC-
TTCAACCATGACGTCCTACGGGAGAAC-ATCAATAAGTC-

AAAACTTTCAACAACGGATCTCTTGGTTCTGGCATCGATGAAGAACGCAGCGAAATGCGATAAGTAATGCGAA
TTGCAGAATTCCGTGAGTCATCGAATCTTTGAACGCACATTGCGCCCTTTGGCATTCCGAAGGGCATGCCTGT
TCGAGCGTCATTATCAACCCTCAAGCCCGGCTTGCTGTTGGGTCC-CACCGTTGG-CGACGATGGTGGACC-
CGAAACATAATGGCAGAGTC-TGCGAGA--CCCTGGATGCAGCGAGCTTCTT-
CAGCACGCATCCCAGCGGCCT-CG-CGGGTCTGTC-TTAACCG-ATCAAAAATTTCTGGTTGACC
Verrucaria_kuusamoensis_MT229774 CATTATCGAG-TTAGGGTCCTCTGGGCCCC-
ATCT-CCAACCC-TTGTCTACC-----ACGTTGCCCC--CGG---CGCTTCTGTCGGGGTCAAC---
TTGAAATTT--------------------------C-TTTTTTAAA-CCC-
TTCAACTATGACGTCCTACGGGAGAGG-ATCAATGAATT-
AAAACTTTCAACAACGGATCTCTTGGTTCTGGCATCGATGAAGAACGCAGCGAAATGCGATAAGTAATGCGAA
TTGCAGAATTCCGTGAGTCATCGAATCTTTGAACGCACATTGCGCCCTTTGGTATTCCGAAGGGCATGCCTGT
TCGAGCGTCATTATCAACCCTCAAGCTCGGCTTGCTGTTGGGTCC-CACCGTTGG-CGACGGCGGTGGACC-
TGAAAGATAATGGCAGAGTC-CGTGAGA--CCCTGGATGCAGCGAGCTTCTT-
TGGCACGCATCGCAACGGCCT-CG-CGGCTCCGGTCTCAACCG-ATCAAATATCATCTGGTTGACC
Verrucaria_pallidomurina_MT127221 CATTATCGAG-TTAGGGTCCTCTGGGCCCC-
ATCT-CCAACCC-TTGCCTACC-----ACGTTG-CCC--CGG---TATCTCTGCCGGGGTAGCTT--
AAAAAATTT--------------------------C-TTTTTTAAA-TTC-
TTCAACCATGACGTCCTACGGGAGAAC-TTGAATAAATT-
AAAACTTTCAACAACGGATCTCTTGGCTCTGGCATCGATGAAGAACGCAGCGAAATGCGATAAGTAATGCGAA
TTGCAGAATTCCGTGAGTCATCGAATCTTTGAACGCACATTGCGCCCTTTGGCATTCCGAAGGGCATGCCTGT
TCGAGCGTCATTATCAACCCTCAAGCCCGGCTTGCTGTTGGATCC-CACCGCCGG-CGACGGCGGTGGATC-
CGAAAGATAATGGCAGAGTC-TGCGAGA--CCCTGGATGCAGCGAGCTTCTT-
CAGCACGCATCTCGGCGGCCT-CG-CGGCTCGGTC-TTAACCA-ATTACAATNNNNNNNNNNNNNN
Verrucaria_deversa_EU553496 CATTATCGAG-TTTGGGTCCTCTGGGCCCC-
ATCT-CCAACCC-TTGCCTACC-----ACGTTG-CCC--CGG---TGTCTCTGCCGGGATAGCTT--
AAAAAATTT--------------------------C--TTTTTAAAATTC-
TTCAACCATGACGTCCTACGGGAGAAT-ATCAATAAATT-
AAAACTTTCAACAACGGATCTCTTGGTTCTGGCATCGATGAAGAACGCAGCGAAATGCGATAAGTAATGCGAA
TTGCAGAATTCCGTGAGTCATCGAATCTTTGAACGCACATTGCGCCCTTTGGTATTCCGAAGGGCATGCCTGT
TCGAGCGTCATTATCAACCCTCAAGCCCGGCTTGCTGTTGGATCC-CACCGTCGG-CGTCGGCGGTGGATC-
CGAAAGATAATGGCAGAGTC-TGCGAGA--CCCTGGATGCAGCGAGCTTCTT-
TAGCACGCATCTCGACGGCTT-CG-CGGCTCGGTC-TTAACCA-ATTACAATCATCNNNNNNNNNN
Verrucaria_sp_MT127205 CATTATCGAG-TTCGGGTCCTCTGGGCCCC-
ATCT-CCAACCC-TTGTCTACC-----ACGTTG-CCC--CGG---TGTCTCTGCCGGGGTCGCTT--
AAAAAATTT--------------------------C-TTTTTTAAA-TTC-
TTCAACCATGACGTCCTACGGGAGAAT-
ATCAATCAATTTAAAACTTTCAACAACGGATCTCTTGGTTCTGGCATCGATGAAGAACGCAGCGAAATGCGAT
AAGTAATGCGAATTGCAGAATTCCGTGAGTCATCGAATCTTTGAACGCACATTGCGCCCTTTGGTATTCCGGA
GGGCATGCCTGTTCGAGCGTCATTATCAACCCTCAAGCCCGGCTTGCTGTTGGATCC-CACCGTCGG-
CGACGACGGTGGATC-CGAAAGATAATGGCAGAGTC-TGCGAGA--CCCTGGATGCAGCGAGCTTCTT-
TAGCACGCATCTCGACGGCCT-TG-CGGCTCGGTCTTTAACCA-ATTATAATCATCCTGGTTGACC
Verrucaria_anziana_FJ664835 CATTATCGAG-TTAGGGTCCTCCGGGCCCC-
ATCTCCCAACCC-TTGCCTACC-----ACGTTG-CCC--GGG---CGTCTCAGCCGGGGTAGCTTA-
AAAAAATTT--------------------------C--TTTTTAAA-
TCCTTTTAACCTTGACGTCCTGCGGGAGAAG-ATCAATCAATT-
AAAACTTTCAACAACGGATCTCTTGGTTCTGGCATCGATGAAGAACGCAGCGAAATGCGATAAGTAATGCGAA
TTGCAGAATTCCGTGAGTCATCGAATCTTTGAACGCACATTGCGCCCTTTGGTATTCCGAAGGGCATGCCTGT
CCGAGCGTCATTATCAACCCTCAAGCCCGGCTTGCTGTTGGATCC-CACCGCCGT-CGCCGGCGGTGGATC-
CGAAAGATAGTGGCAGAGTC-TGCGAGA--CCCTGGATGCAGCGAGCTT-TT-
CGGCACGCATCTCAACGGCCT-CG-CGGCTCGGTC-TTAACCG-ATCAAA-TTCATCTGGTTGACC
Verrucaria_karelica_MT229762 CATTATCGAG-
TTAGGGTCCTCTGGGCCCCAATCT-CCAACCC-CTGTCTACC-----AAGTCG-CCC--CGG---
CGCTTCTGCCGGGGTGACATTTTAAAAATTT--------------------------C--TTTTTAAA-TCC-
TTCAACCATGACGTCCTACGGGAGAAA-ATCAATCAATT-
AAAACTTTCAACAACGGATCTCTTGGTTCTGGCATCGATGAAGAACGCAGCGAAATGCGATAAGTAATGCGAA
TTGCAGAATTCCGTGAGTCATCGAATCTTTGAACGCACATTGCGCCCTTTGGTATTCCGAAGGGCATGCCTGT
CCGAGCGTCATTATCAACCCTCAAGCCCGGCTTGCTGTTGGATCT-CACCGTCGG-CGACGGTGGTGGATC-

CCAAAGATAATGGCAGAGTC-TGCGAGA--CCCTGGATGCAGCGAGCTTCTT-
CAGCACGCATCGCAACGGCCT-CG-CGGCTCGGCC-TCAACCG-ACGAAATTTCATCTGGTTGACC
Verrucaria_devergens_MT229741 CATTATCGAG-TTAGGGTCCTCTGGGCCCC-
ATCT-CCCACCC-TTGCCTACC-----ACGTCG-CCC--CGG---CGCTTCTGCTGGGGTGATTT--
TAAAAATTT--------------------------C-TTTTTTAAA-TTC-
TTCAACCATGACGTCCTACGGGAGAGA-ATCAATCAATT-
AAAACTTTCAACAACGGATCTCTTGGTTCTGGCATCGATGAAGAACGCAGCGAAATGCGATAAGTAATGCGAA
TTGCAGAATTCCGTGAGTCATCGAATCTTTGAACGCACATTGCGCCCTTTGGTATTCCGAAGGGCATGTCTGT
CCGAGCGTCATTATCAACCCTCAAGCCCGGCTTGCTGTTGGATCT-CACCGTTGG-CGACGGCGGTGGATC-
CCAAAGATAATGGCAGAGTC-TGCGAGA--CCCTGGATGCAGCGAGCTTCTT-
CAGCACGCATCGCAACGGCCT-CG-CGGCTCGGCC-TCAACCG-ACGAAATTTCATCTGGTTGACC
Verrucaria_subdevergens_MT229782 CATTATCGAG-TTAGGGTCCTCTGGGCCTC-
ATCT-CCAACCC-TTGTCTACC-----ACGTCG-CCC--CGG---CGCTTCTGCCGGGGTGATTT--
TAAAAATTT--------------------------C--TTTTTAAA-TTC-
TTCAACCATGACGTCCTACGGGAGAGA-
ATCAATCAATTTAAAACTTTCAACAACGGATCTCTTGGTTCTGGCATCGATGAAGAACGCAGCGAAATGCGAT
AAGTAATGCGAATTGCAGAATTCCGTGAGTCATCGAATCTTTGAACGCACATTGCGCCCTTTGGTATTCCGAA
GGGCATGCCTGTCCGAGCGTCATTATCAACCCTCAAGCCCGGCTTGCTGTTGGATCT-CACCGTTGG-
CGACGGCGGTGGATC-CCAAAGATAATGGCAGAGTC-TGCGAGA--CCCTGGATGCAGCGAGCTTCTT-
TAGCACGCATCGCAACGGCCT-CG-CGGCTCGGCC-TGAACCG-ACGAAATTTCATCTGGTTGACC
Verrucaria_subtilis_MT229810 CATTATCGAG-TTAGGGTCCTCCGGGCCCC-
ATCT-CCAACCC-TTGTCTACT-----ACGTCG-CCT--CGG---CGCTTCCGCTGGGGTGACT---
TAAAAATTT--------------------------C--TTTTTAAA-TTC-
TTTAACCATGACGTCCTGCGGGAGAAAAACCAATCAATT-
AAAACTTTCAACAACGGATCTCTTGGTTCTGGCATCGATGAAGAACGCAGCGAAATGCGATAAGTAATGCGAA
TTGCAGAATTCCGTGAGTCATCGAATCTTTGAACGCACATTGCGCCCTTTGGTATTCCGAAGGGCATGCCTGT
TCGAGCGTCATTATCAACCCTCAAGCCCGGCTTGCTGTTGGATCT-CACCGTTGG-CGACGGCGGTGGATC-
CGAAAGATAATGGCAGAGCC-TGTGAGA-CCCCTGGATGCAGCGAGCTTCTT-
CAGCACGCATCGCAACGGCCT-CG-CGGCTCGGTC-TTAACCG-ACAAAA-TTCATCTGGTTGACC
Verrucaria_cavernarum_MT229724 CATTATCGAGTTTAGGGTCCTCCGGGCCCC-
ATCT-CCAACCC-TTGTCTACC-----ACATCG-CCC--CGG---CGTCTCTGCTGGGGTGACTTT-
TAAAAATTT--------------------------C--TTTTTAAA-TTC-
TTCAACCATGACGTCCTGCGGGAGACGAATCAATCAATT-
AAAACTTTCAACAACGGATCTCTTGGTTCTGGCATCGATGAAGAACGCAGCGAAATGCGATAAGTAATGCGAA
TTGCAGAATTCCGTGAGTCATCGAATCTTTGAACGCACATTGCGCCCTTTGGTATTCCGAAGGGCATGCCTGT
TCGAGCGTCATTATCAACCCTCAAGCCCGGCTTGCTGTTGGATCT-CACCGTTGG-CGACGGCGGTGGATC-
CGAAAGATAATGGCAGAGTC-TGTGAGA-CCTCTGGATGCAGCGAGCTTCTT-
CAGCACGCATCGCAACGGCCT-CG-CGGCTCGGTC-TCAACCG-ACAAAATTTCATCTGGTTGACC
Verrucaria_calkinsiana_KT695332 CATTATCGAG-TTAGGGTCCTCCGGGCCCC-
ATCT-CCAACCC-TTGTCTACC-----ACGTCG-CCC--CGG---CGCTTCGGCCGGGGCGACTTT-
TGAAAATTT--------------------------C--CTTTTAAA-TTC-
TTCAACCATGACGTCCTGCGGGAGAAAAATCAATCAATC-
AAAACTTTCAACAACGGATCTCTTGGTTCTGGCATCGATGAAGAACGCAGCGAAATGCGATAAGTAATGCGAA
TTGCAGAATTCCGTGAGTCATCGAATCTTTGAACGCACATTGCGCCCTTTGGTATTCCGAAGGGCATGCCTGT
TCGAGCGTCATTATCAACCCTCAAGCCCGGCTTGCTGTTGGATCT-CGCCGCTGG-CGACGGCGGTGGATC-
CGAAAGATAATGGCAGAGTC-TGTGAGACCCCCTGGATGCAGCGAGCTTCTT-
CAGCACGCATCGCAATGGCCT-CG-CGGCTCGGTC-TTAACCG-ACAAAATTTCATCTGGTTGACC
Verrucaria_bifurcata_MT229720 CATTATCGAG-TTAGGGTCCTCCGGGCCCC-
ACCT-CCAACCC-TTGTCTACC-----ACGTCG-CCC--CGG---CGCTTCTGCCGGGGTGACTTA-
AAAAAATTT--------------------------C--TTTTTAAA-TTC-
TTCAACCATGACGTCCTGCGGGAGAATAATCAATCAGTT-
AAAACTTTCAACAACGGATCTCTTGGTTCTGGCATCGATGAAGAACGCAGCGAAATGCGATAAGTAATGCGAA
TTGCAGAATTCCGTGAGTCATCGAATCTTTGAACGCACATTGCGCCCTTTGGTATTCCGAAGGGCATGCCTGT
TCGAGCGTCATTATCAACCCTCAAGCCCGGCTTGCTGTTGGGTCT-CACCGCTGG-CGACGGCGGTGGATC-
CGAAAGATAATGGC--AGTC-TGTGAGA-CCCCTGGATGTAGCGAGCTTCTT-
CAGCACGCATCGCAACGGCCT-CG-CGGCTCGGTC-TTAACCG-ACAAAATTTCATCTGGTTGACC
Verrucaria_difficilis_MT229743 CATTATCGAG-TTAGGGTCCTCCGGGCCCC-
ATCT-CCAACCC-TTGTCTACC-----ACGTCG-CCC--CGG---

CGCTTCTGCCGGGGTGACTTGTGAAAAATTT-------------------------C--TTTTTAAA-TTC-
TTCAACCATGACGTCCTGCGGGAGAAAAATCAATCAATT-
AAAACTTTCAACAACGGATCTCTTGGTTCTGGCATCGATGAAGAACGCAGCGAAATGCGATAAGTAATGCGAA
TTGCAGAATTCCGTGAGTCATCGAATCTTTGAACGCACATTGCGCCCTTTGGTATTCCGAAGGGCATGCCTGT
TCGAGCGTCATTATCAACCCTCAAGCCCGGCTTGCTGTTGGATCT-CACCGCTGG-CGACGGCGGTGGATC-
CGAAAGATAATGGCAGAGTC-TGTGAGA-CCTCTGGATGCAGCGAGCTTCTT-
CAGCACGCATCGCAACGGCCT-CG-CGGCTCGGTC-TTAACCG-ACAAAATTTCATCTGGTTGACC
Polyblastia_sp_EU553519 CATTATCGAG-TTAGGGTCCTCTGGGTCCC-
ACCT-CCAACCC-TTGTCTACC-----ACGTTG-CCC--CGG---CGCTCCTGCCGGGGCGACTTT-
TGAAAATTT-------------------------C-TTTTTTAAA-TTC-
TTTAACCGTGTCGTCCTACGGGAGATGAAGCAATATAT--
AAAACTTTCAACAACGGATCTCTTGGTTCTGGCATCGATGAAGAACGCAGCGAAATGCGATAAGTAATGCGAA
TTGCAGAATTCCGTGAGTCATCGAATCTTTGAACGCACATTGCGCCCTTTGGTATTCCGAAGGGCATGCCTGT
CCGAGCGTCATTATCAACCCTCAAGCCCGGCTTGCTGTTGGGTCC-CACCGTCGG-CGACGGCGGTGGACC-
CGAAAGAGAATGGCAGAGTC-TACCGGA--CCCCGGATGCAGCGAGCTTCCT-
CAGCACGCATCCTAGGGGCCT-CG-GGGCGCTGTC-TTAACCA-ATAAA--TTAATCTNNNNNNNN
Thelidium_sp2_52038 CATTATCGAG-TTAGGGTCCTCTGGGCCCC-
ATCT-CCAACCC-TTGTCTACC-----ACGTCG-CCC--CGG---CGCTTCTGCCGGGGAGACATT-
TGAAAATTT-------------------------C--TTTTTAAA-TCC-
TTCAACCATGACGTCCTACGGGAGAAT-
AACAATCAATTAAAAACTTTCAACAACGGATCTCTTGGTTCTGGCATCGATGAAGAACGCAGCGAAATGCGAT
AAGTAATGCGAATTGCAGAATTCCGTGAGTCATCGAATCTTTGAACGCACATTGCGCCCTTTGGTATTCCGAA
GGGCATGCCTGTCCGAGCGTCATTATCAACCCTCAAGCCCGGCTTGCTGTTGGGTCC-CACCGTCGG-
CAACGGCGGTGGACC-CGAAAGATAATGGCAGAGTC-TGTGAGA--CCCTGGATGCAGCGAGCTTCCT-
CAGCACGCATCGTAAGGGCCT-CG-GGGCGCTGTC-TTAACCA-AT-----TTCATCTGGTTGACC
Thelidium_incavatum_42871 CATTATCGAG-TTAGGGTCTTCTGGGCCCC-
ATCT-CCAACCC-TTGTCTACC-----ACGTCG-CTC-------------CTGCCGGGGCGGCT---
TGAAAATTT-------------------------C-TTTTTTAAA-TTC-
GTCAACCATGACGTCCTACGGGAGAAT-ATCAATCAATC-
AAAACTTTCAACAACGGATCTCTTGGTTCTGGCATCGATGAAGAACGCAGCGAAATGCGATAAGTAATGCGAA
TTGCAGAATTCCGTGAGTCATCGAATCTTTGAACGCACATTGCGCCCTTTGGTATTCCGAAGGGCATGCCTGT
TCGAGCGTCATTATCAACCCTCAAGCCCGGCTTGCTGTTGGGTCT-CATCGTCGG-CGACGGCGGTGGACC-
CGAAAGATAACGGCAGAGTC-CGCGAGA--CCCTGGATGCAGCGAGCTTCCT-
CAGCACGCATCCTGGCGGCCT-CGGGGGCGTTGTC-TCACCCA-ACAACA---CATCTGGTTGACC
Thelidium_incavatum_38227 CATTATCGAG-TTAGGGTCTTCTGGGCCCC-
ATCT-CCAACCC-TTGTCTACC-----ACGTCG-CTC-------------CTGCCGGGGCGACT---
TGAAAATTT-------------------------C-TTTTTTAAA-TTC-
TTCAACCATGACGTCCTACGGGAGAAT-ATCAATCAATC-
AAAACTTTCAACAACGGATCTCTTGGTTCTGGCATCGATGAAGAACGCAGCGAAATGCGATAAGTAATGCGAA
TTGCAGAATTCCGTGAGTCATCGAATCTTTGAACGCACATTGCGCCCTTTGGTATTCCGAAGGGCATGCCTGT
TCGAGCGTCATTATCAACCCTCAAGCCCGGCTTGCTGTTGGGTCT-CACCGTCGG-CGACGGCGGTGGACC-
CGAAAGATAATGGCAGAGTC-CGCGAGA--CCCTGGATGCAGCGAGCTTCCT-
TAGCACGCGTCCTGGCGGCCT-CGCGGGCGCTGTC-TCACCCA-CTAACA---CATCTGG-TGACC
Thelidium_incavatum_34722 CATTATCGAG-TTAGGGTCTTCTGGGCCCC-
ATCT-CCAACCC-TTGTCTACC-----ACGTCG-CTC-------------CTGCCGGGGCGACT---
TGAAAATTT-------------------------C-TTTTTTAAA-TTC-
TTCAACCATGACGTCCTACGGGAGAAT-ATCAATCAATC-
AAAACTTTCAACAACGGATCTCTTGGTTCTGGCATCGATGAAGAACGCAGCGAAATGCGATAAGTAATGCGAA
TTGCAGAATTCCGTGAGTCATCGAATCTTTGAACGCACATTGCGCCCTTTGGTATTCCGAAGGGCATGCCTGT
TCGAGCGTCATTATCAACCCTCAAGCCCGGCTTGCTGTTGGGTCT-CACCGTCGG-CGACGGCGGTGGACC-
CGAAAGATAATGGCAGAGTC-CGCGAGA--CCCTGGATGCAGCGAGCTTCCT-
TAGCACGCGTCCTGGCGGCCTCCGCGGGCGCTGTC-TCACCCA-CTAACA---CATCTGGTTGACC
Thelidium_incavatum_35282 NNNNATCGAG-TTAGGGTCTTCTGGGCCCC-
ATCT-CCAACCC-TTGTCTACCC----ACGTCG-CTC-------------CTGCCGGGGCGACT---
TGAAAATTT-------------------------C-TTTTTTAAA-TTC-
TTCAACCATGACGTCCTACGGGAGAAT-ATCAATCAATC-
AAAACTTTCAACAACGGATCTCTTGGTTCTGGCATCGATGAAGAACGCAGCGAAATGCGATAAGTAATGCGAA
TTGCAGAATTCCGTGAGTCATCGAATCTTTGAACGCACATTGCGCCCTTTGGTATTCCGAAGGGCATGCCTGT

TCGAGCGTCATTATCAACCCTCAAGCCCGGCTTGCTGTTGGGTCT-CACCGTCGG-CGACGGCGGTGGACC-
CGAAAGATAATGGCAGAGTC-CGCGAGA--CCCTGGATGCAGCGAGCTTCCT-
CAGCACGCGTCCTGGCGGCCT-CGGGGGCGCTGTC-TCACCCA-ACAACA---CATCTGGTTGACC
Thelidium_incavatum_36857   CATTATCGAG-TTAGGGTCTTCTGGCCCC-
ATCT-CCAACCC-TTGTCTACCC----ACGTCG-CTC-------------CTGCCGGGGCGACT---
TGAAAATTT--------------------------C-TTTTTTAAA-TTC-
TTCAACCATGACGTCCTACGGGAGAAT-ATCAATCAATC-
AAAACTTTCAACAACGGATCTCTTGGTTCTGGCATCGATGAAGAACGCAGCGAAATGCGATAAGTAATGCGAA
TTGCAGAATTCCGTGAGTCATCGAATCTTTGAACGCACATTGCGCCCTTTGGTATTCCGAAGGGCATGCCTGT
TCGAGCGTCATTATCAACCCTCAAGCCCGGCTTGCTGTTGGGTCT-CACCGTCGG-CGACGGCGGTGGACC-
CGAAAGATAATGGCAGAGTC-CGCGAGA--CCCTGGATGCAGCGAGCTTCCT-
CAGCACGCGTCCTGGCGGCCT-CGGGGGCGCTGTC-TCACCCA-ACAACA---CATCTGGTTGACC
Polyblastia_sp_EU553503   CATTATCGAG-TTAGGGTCTTCTGGCCCC-
ATCT-CCAACCC-TTGTCTACC-----ACGTCG-CTC-------------CTGCCGGGGCGACT---
TGAAAATTT--------------------------C--TTTTTTAA-TTC-
TTCAACCATGACGTCCTACGGGAAAAT-ATCAATCAATC-
AAAACTTTCAACAACGGATCTCTTGGTTCTGGCATCGATGAAGAACGCAGCGAAATGCGATAAGTAATGCGAA
TTGCAGAATTCCGTGAGTCATCGAATCTTTGAACGCACATTGCGCCCTTTGGTATTCCGAAGGGCATGCCTGT
TCGAGCGTCATTATCAACCCTCAAGCCCGGCTTGCTGTTGGGTCT-CACCGTCGG-CGACGGCGGTGGACC-
CGAAAGATAATGGCAGAGTC-CGCGAGA--CCCTGGATGCAGCGAGCTTCCT-
CAGCACGCGTCCTGGCGGCCT-CGCGGGCGCTGTC-TCACCCA-ACAACA---CATCNNNNNNNNN
Thelidium_incavatum_38399   CATTATCGAG-TTAGGGTCTTCTGGCCCC-
ATCT-CCAACCC-TTGTCTACC-----ACGTCG-CTC-------------CTGCCGGGGCGACT---
TGAAAATTT--------------------------C--TTTTTTAA-TTC-
TTCAACCATGACGTCCTACGGGAAAAT-ATCAATCAATC-
AAAACTTTCAACAACGGATCTCTTGGTTCTGGCATCGATGAAGAACGCAGCGAAATGCGATAAGTAATGCGAA
TTGCAGAATTCCGTGAGTCATCGAATCTTTGAACGCACATTGCGCCCTTTGGTATTCCGAAGGGCATGCCTGT
TCGAGCGTCATTATCAACCCTCAAGCCCGGCTTGCTGTTGGGTCT-CACCGTCGG-CGACGGCGGTGGACC-
CGAAAGATAATGGCAGAGTC-CGCGAGA--CCCTGGATGCAGCGAGCTTCCT-
CAGCACGCGTCCTGGCGGCCT-CGCGGGCGCTGTC-TCACCCA-ACAACA---CATCTGG-TGACC
Thelidium_incavatum_36867   NNNNATCGAG-TTAGGGTCTTCTGGCCCC-
ATCT-CCAACCC-TTGTCTACC-----ACGTCG-CTC-------------CTGCCGGGGCGACT---
TGAAAATTT--------------------------C-TTTTTTAAA-TTC-
TTCAACCATGACGTCCTACGGGAAAAT-ATCAATCAATC-
AAAACTTTCAACAACGGATCTCTTGGTTCTGGCATCGATGAAGAACGCAGCGAAATGCGATAAGTAATGCGAA
TTGCAGAATTCCGTGAGTCATCGAATCTTTGAACGCACATTGCGCCCTTTGGTATTCCGAAGGGCATGCCTGT
TCGAGCGTCATTATCAACCCTCAAGCCCGGCTTGCTGTTGGGTCT-CACCGTCGG-CGACGGCGGTGGACC-
CGAAAGATAATGGCAGAGTC-CGCGAGA--CCCTGGATGCAGCGAGCTTCCT-
CAGCACGCGTCCTGGCGGCCT-CGCGGGCGCTGTC-TCACCCA-ACAACA---CATCTGGTTGACC
Thelidium_incavatum_46459   CATTATCGAG-TTAGGGTCTTCTGGCCCC-
ATCT-CCAACCC-TTGTCTACC-----ACGTCG-CTC-------------CTGCCGGGGCGACT---
TGAAAATTT--------------------------C-TTTTTTAAA-TTC-
TTCAACCATGACGTCCTACGGGAAAAT-ATCAATCAATC-
AAAACTTTCAACAACGGATCTCTTGGTTCTGGCATCGATGAAGAACGCAGCGAAATGCGATAAGTAATGCGAA
TTGCAGAATTCCGTGAGTCATCGAATCTTTGAACGCACATTGCGCCCTTTGGTATTCCGAAGGGCATGCCTGT
TCGAGCGTCATTATCAACCCTCAAGCCCGGCTTGCTGTTGGGTCT-CACCGTCGG-CGACGGCGGTGGACC-
CGAAAGATAATGGCAGAGTC-CGCGAGA--CCCTGGATGCAGCGAGCTTCCT-
CAGCACGCGTCCTGGCGGCCT-CGCGGGCGCTGTC-TCACCCA-ACAACA---CATCTGGTTGACC
Thelidium_incavatum_37971   CATTATCGAG-TTAGGGTCTTCTGGCCCC-
ATCT-CCAACCC-TTGTCTACC-----ACGTCG-CTC-------------CTGCCGGGGCGACT---
TGAAAATTT--------------------------C-TTTTTTAAA-TTC-
TTCAACCATGACGTCCTACGGGAAAAT-ATCAATCAATC-
AAAACTTTCAACAACGGATCTCTTGGTTCTGGCATCGATGAAGAACGCAGCGAAATGCGATAAGTAATGCGAA
TTGCAGAATTCCGTGAGTCATCGAATCTTTGAACGCACATTGCGCCCTTTGGTATTCCGAAGGGCATGCCTGT
TCGAGCGTCATTATCAACCCTCAAGCCCGGCTTGCTGTTGGGTCT-CACCGTCGG-CGACGGCGGTGGACC-
CGAAAGATAATGGCAGAGTC-CGCGAGA--CCCTGGATGCAGCGAGCTTCCT-
CAGCACGCGTCCTGGCGGCCT-CGCGGGCGCTGTC-TCACCCA-ACAACA---CATCTGGTTGACC
;

```
END;
```

[End of File]
